# Supplementary material for: Intrareticular charge transfer regulated electrochemiluminescence of donor–acceptor covalent organic frameworks
Source: Nat Commun. 2021 Nov 23;12:6808. doi: 10.1038/s41467-021-27127-5 (PMC8611053; doi:10.1038/s41467-021-27127-5)
Supplement: Supplementary file 1 — Supplementary Information [file 41467_2021_27127_MOESM1_ESM.pdf]

Supplementary Information

Intrareticular charge transfer regulated electrochemiluminescence of donor-acceptor covalent organic frameworks

Rengan Luo[1,4], Haifeng Lv[2,4], Qiaobo Liao[3], Ningning Wang[1], Jiarui Yang[1], Yang Li[1] , Kai Xi[3]*, Xiaojun Wu[2]*, Huangxian Ju[1] & Jianping Lei[1]*

[1]State Key Laboratory of Analytical Chemistry for Life Science, School of Chemistry and Chemical Engineering, Nanjing University, Nanjing 210023, China. [2]Hefei National Laboratory for Physical Sciences at the Microscale, Synergetic Innovation of Quantum Information and Quantum Technology, CAS Center for Excellence in Nanoscience, and School of Chemistry and Materials Sciences, University of Science and Technology of China, Hefei 230026, China. [3]School of Chemistry and Chemical Engineering, Nanjing University, Nanjing 210023, China. [4]These authors contributed equally: Rengan Luo, Haifeng Lv. *e-mail: xikai@nju.edu.cn; xjwu@ustc.edu.cn; jpl@nju.edu.cn

Supporting Methods

Reagents. Tris(4-formylphenyl)amine (TFPA, 98%) and 1,3,5-Tris(4-aminophenyl)benzene (TAPB, 98%) were obtained from Jilin Chinese Academy of Sciences-Yanshen Technology Co., Ltd. Tris(4-aminophenyl)amine (TAPA, 98%) and aniline (99.5%) were purchased from Shanghai Macklin Biochemical Co., Ltd. 2,4,6-Tris(4-aminophenyl)-1,3,5-triazine (TAPT, 95%) was received from Heowns Biochem. Acetic acid (99.5%), triethylamine (TEtA, 98%), and tri-n-propylamine (TPrA, 98%) were obtained from Shanghai J&K Scientific Ltd. 1,4-Dioxane (99.5%) and tetrahydrofuran (THF, 99%) were bought from Nanjing Chemical Reagent Co., Ltd. 1,2-dichlorobenzene (o-DCB, 99%) was obtained from Shanghai Aladdin Bio-Chem Technology Co., Ltd. n-Butanol (99.8%), benzaldehyde (98%), and 1,3,5-trimethylbenzene (97%) were purchased from Shanghai Meryer Chemical Technology Co., Ltd. Potassium dihydrogen phosphate (99.5%), potassium phosphate dibasic trihydrate (99.0%), acetonitrile (99%), ferrocene (99.5%), and tetrabutylammonium hexafluorophosphate (TBAPF6, 98%) were obtained from Shanghai Titan Scientific Co., Ltd. Phosphate buffer solutions (PBS, 0.10 M, pH = 6.0–8.0) were prepared by mixing stock solutions of KH_2PO_4 and K_2HPO_4. All the reagents are used as received without further purification.

Powder X-ray Diffraction. Powder X-ray diffraction (PXRD) patterns were collected on Bruker D8 Advance employing Cu Kα line focused radiation at 40 kV, 40 mA powder. Samples were placed on a silicon zero background sample holder, and then the sample surface was leveled with a clean microscope slide. No sample grinding was used prior to analysis unless specifically mentioned. Samples were rotated as diffraction data were collected using a continuous 2θ scan from 3–50°.

Gas Sorption Analysis. Surface areas were measured by nitrogen sorption at 77.3 K. The powder samples were degassed under vacuum at 120 °C for 4–6 h. Isotherm measurements were performed using a JW-BK200B volumetric gas sorption instrument equipped with nitrogen containers.

NMR Spectrum. Liquid NMR spectra were recorded on Bruker Advance 600 or III at 600 MHz or 400 MHz for ^{1}H and 150 MHz or 100 MHz for ^{13}C nuclei. Meanwhile, solid-state NMR experiments were performed on a Bruker Advance NEO 600 MHz spectrometer. The ^{13}C cross-polarization magic-angle spinning (^{13}C CP/MAS NMR) spectra were recorded with a 4-mm MAS probe and a sample spinning rate of 14.0 kHz.

Spectral Measurement. Fourier-transform infrared (FT-IR) spectra were collected using a Nicolet iS FT-IR in ATR mode. Photoluminescence (PL) spectra were collected on Edinburgh FLS980 using a quartz cell. The cell weights of *t*-COF, *b*-COF, and *a*-COF were regarded as their molar weight, which were 629.722 g mol^{-1}, 626.759 g mol^{-1}, and 565.676 g mol^{-1}, respectively. Solid-state Ultraviolet-visible (UV-Vis) spectra of COF samples were recorded on Shimadzu UV-3600 in a

diffuse reflection mode. The ultraviolet photoelectron spectroscopy (UPS) spectra were measured using ESCALAB250Xi instrument with a monochromatic He I light source (21.22 eV).

Electron Microscopy. Transmission electron microscopy (TEM) images were collected using a JEOL JEM-2800 transmission electron microscopy, operated at an acceleration voltage of 200 kV. On the other hand, scanning electron microscopy (SEM) images were recorded using a JEOL JSM-7800F field emission scanning electron microscope. Samples for SEM test were prepared by depositing the dry powders on a silicon disk and then coating the samples with Au before measurements.

ECL Imaging. The ECL imaging system was equipped with a classical three-electrode system containing a modified carbon/ITO as working electrode (10 μL of 1.0 mg mL^{-1} COF/1,4-dioxane suspension was drop-coated on the surface), a platinum wire as counter electrode, and a Ag/AgCl electrode as reference electrode. ECL images were recorded by applying a constant potential of 1.40 V for 10 s in 0.10 M PBS (pH = 6.8) containing 0.10 M TPrA solution. ECL images were captured through a homemade multicolor ECL imaging system equipped with a Canon focus lens (EF 50 mm f/1.2 L USM) and a QImaging Retiga R6 color scientific CCD camera in a dark box.[1] The relative ECL efficiency of *t*-COF with respect to 1.0 μM $Ru(bpy)_3^{2+}$ was calculated to be 23.2% in 0.10 M PBS (pH = 6.8) containing 20 mM TPrA.[2]

ECL Spectrum. ECL spectra were recorded on a homemade ECL spectrum analyzer consisting of a Princeton Acton P-2300 monochromator equipped with a grating (grating density: 50 g mm^{-1}; blazed wavelength: 600 nm), a liquid N_2 cooled Princeton PyLoN digital charge-coupled device (CCD) detector, and a CHI-660D electrochemical workstation.

Synthesis of Model Compounds.

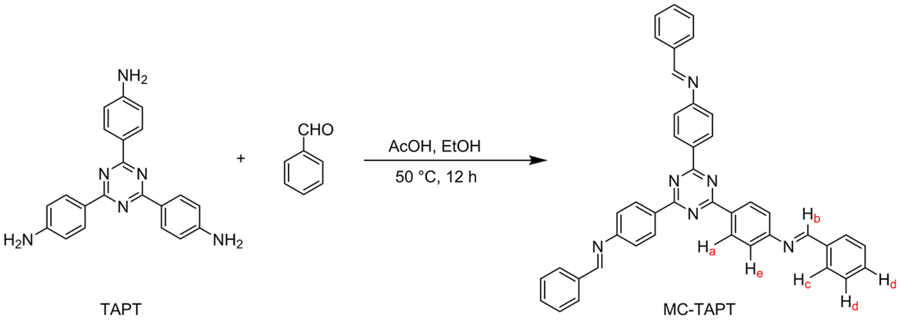

Model compounds were synthesized according to a reported procedure with some modifications.[3] First, acetic acid (19 μL, 0.1 equiv.) was added to a light yellow mixture of TAPT (354 mg, 1.0 mmol) and benzaldehyde (337 μL, 3.3 mmol) in ethanol (10 mL). The reaction was then stirred at 50 °C for 12 h. When cooled to room temperature, the solid was collected by filtration and thrice washed with ice-cold ethanol (10 mL). The pure yellow product was obtained after removing the

solvent under vacuum at room temperature to afford MC-TAPT in a yield of 480 mg: 78%. ^{1}H NMR (DMSO-d_6, 400 MHz): δ = 8.8–8.81 (d, 6H_a), 8.74 (s, 3H_b), 8.03–8.01 (m, 6H_c), 7.60–7.57 (m, 9H_d), 7.52–7.50 (d, 6H_e). ^{13}C NMR ($CDCl_3$, 150 MHz): δ = 170.81, 161.13, 155.67, 135.94, 133.79, 131.72, 130.11, 129.06, 128.81, 121.00. HRMS (m/z) $[M+H]^+$, $[C_{42}H_{30}N_6+H]^+$: Calc. 619.2605, Found 619.2616.

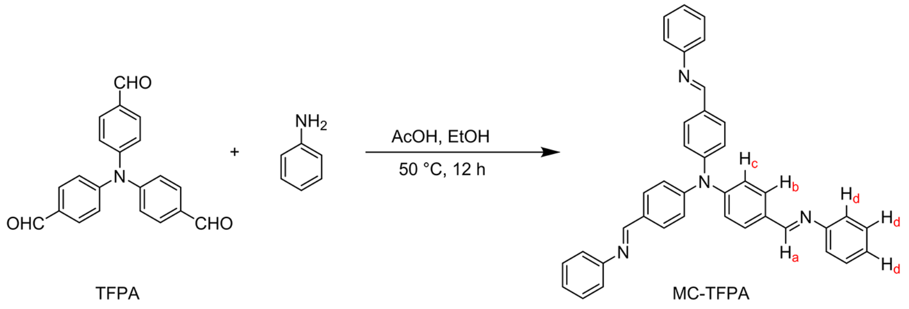

Similarly, acetic acid (19 µL, 0.1 equiv.) was added to the yellow mixture of TFPA (329 mg, 1.0 mmol) and aniline (301 µL, 3.3 mmol) in ethanol (10 mL). Orange MC-TFPA was obtained in a yield of 471 mg: 85%. ^{1}H NMR (DMSO-d_6, 400 MHz): δ = 8.58 (s, 3H_a), 7.95–7.93 (d, 6H_b), 7.44–7.40 (m, 6H_c), 7.26–7.22 (m, 15H_d). ^{13}C NMR (DMSO-d_6, 100 MHz): δ = 160.10, 152.13, 149.28, 132.09, 130.80, 129.68, 126.26, 124.58, 121.44. HRMS (m/z) $[M+H]^+$, $[C_{39}H_{30}N_4+H]^+$: Calc. 555.2543, Found 555.2545.

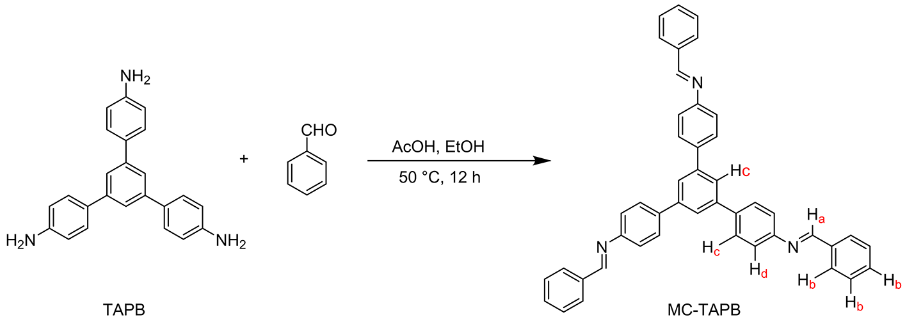

Acetic acid (19 µL, 0.1 equiv.) was added to a white mixture TAPB (351 mg, 1.0 mmol) and benzaldehyde (337 µL, 3.3 mmol) in ethanol (10 mL). Light yellow MC-TAPB was obtained in a yield of 450 mg: 73%. ^{1}H NMR (DMSO-d_6, 400 MHz): δ = 8.74 (s, 3H_a), 8.01–7.97 (m, 15H_b), 7.57–7.55 (m, 9H_c), 7.45–7.43 (d, 3H_d). ^{13}C NMR (DMSO-d_6, 100 MHz): δ = 161.19, 151.38, 141.58, 138.31, 136.54, 132.03, 129.33, 129.24, 128.52, 124.35, 122.15. HRMS (m/z) $[M+H]^+$, $[C_{45}H_{33}N_3+H]^+$: Calc. 616.2747, Found 616.2764.

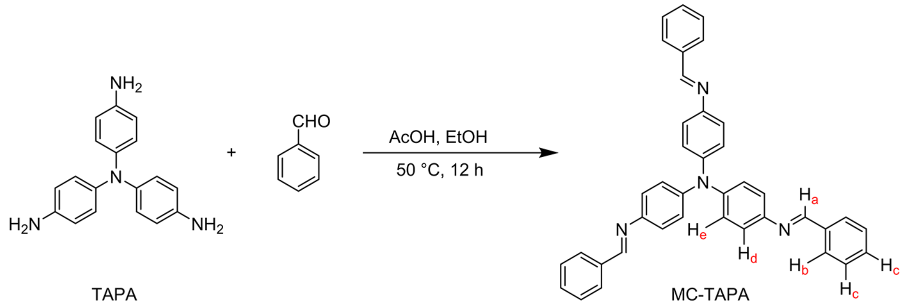

Acetic acid (19 µL, 0.1 equiv.) was added to a purple mixture of TAPA (290 mg, 1.0 mmol) and benzaldehyde (337 µL, 3.3 mmol) in ethanol (10 mL). Dark red MC-TAPA was obtained in a yield of 516 mg: 93%. ^{1}H NMR (DMSO-d_6, 400 MHz): δ = 8.68 (s, 3H_a), 7.95–7.92 (m, 6H_b), 7.54–7.51 (m, 9H_c), 7.33–7.30 (m, 6H_d), 7.13–7.09 (m, 6H_e). ^{13}C NMR (DMSO-d_6, 100 MHz): δ = 159.65, 146.64, 145.86, 136.71, 131.73, 129.29, 128.99, 124.78, 123.02. HRMS (m/z) $[M+H]^+$, $[C_{39}H_{30}N_4+H]^+$: Calc. 555.2543, Found 555.2571.

Supplementary Figures

Structures of Bulk and 2D *t*-, *b*- and *a*-COF

The structures of *a*-, *b*- and *t*-COFs were modelled referring to the similar covalent organic framework crystal structure (TPA-COF-2).[4] The optimized lattice parameters of bulk *a*-COF were a = b = 20.92 Å, c= 3.81 Å; for bulk *b*-COF, a = b = 23.55 Å, c = 17.20 Å; and for bulk *t*-COF, a = b = 23.27 Å, c = 15.70 Å. The lattice parameters of 2D *a*-COF were a = b = 20.85 Å; for 2D *b*-COF, a = b = 23.45 Å; for 2D *t*-COF, a = b = 23.19 Å. The bulk *a*-, *b*- and *t*-COFs were used to simulate the PXRD pattern and 2D *a*-, *b*- and *t*-COFs were used to calculate the electronic properties including the band structures, work functions and energy levels. For bulk *a*-, *b*- and *t*-COFs, we adopt the AA-stacking, in which the interlayer distance of *a*-, *b*- and *t*-COFs was 3.81 Å, 3.77 Å and 3.71 Å, respectively. The unit-cell of bulk and 2D *a*-COF consists of 39 carbon atoms (C), 27 hydrogen atoms (H) and 5 nitrogen atoms (N). The unit-cell of bulk and 2D *b*-COF consists of 45 carbon atoms, 30 hydrogen atoms and 4 nitrogen atoms. The unit-cell of bulk and 2D *t*-COF consists of 42 carbon atoms, 27 hydrogen atoms and 7 nitrogen atoms. The structural difference of *a*-, *b*- and *t*-COF lies in the central nodes, in which the *a*-COF owns two nitrogen atoms; *b*-COF owns one benzene ring and one nitrogen atom; *t*-COF owns one triazine ring and one nitrogen atom. The Pawley PXRD refinements were performed using the Reflex module in the Materials Studio 7.0, in which the Thompson-Cox-Hastings profile functions and Finger-Cox-Jephcoat corrections were used for the profile fitting

(peak broadening, peak asymmetry and zero shift error were taken into account).

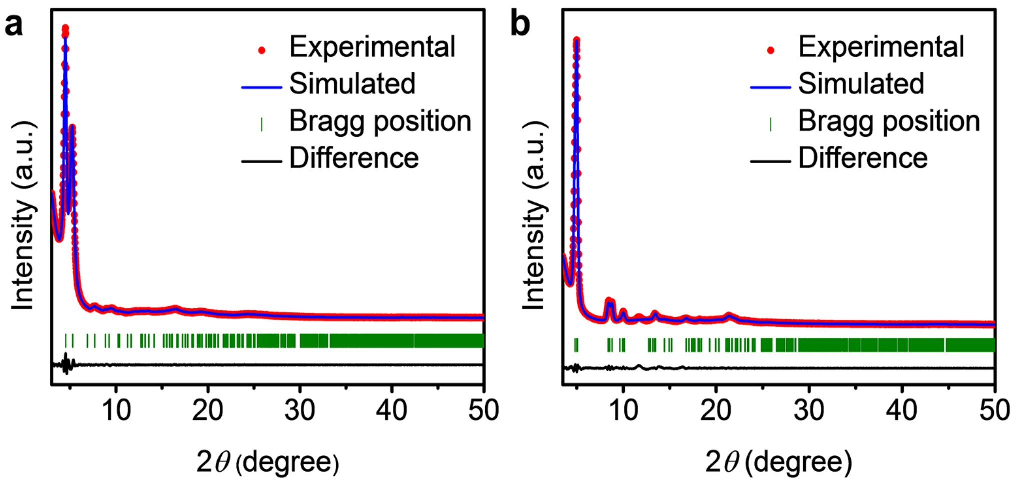

Supplementary Figure 1 | PXRD of *b*-COF and *a*-COF. (**a**) Experimental (red dot) and simulated (blue line) PXRD patterns of *b*-COF in eclipsed stacking mode. Unweighted-profile R factor (Rp) = 1.93% and weighted-profile R factor (Rwp) = 2.54%. Powley-refined triclinic unit cell parameters are: a = 23.46845 Å, b = 23.43171 Å, and c = 17.25418 Å; α = 90.00000°, β = 90.00000°, and γ = 119.99866°. (**b**) Experimental (red dot) and simulated (blue line) PXRD patterns of *a*-COF in eclipsed stacking mode. Unweighted-profile R factor (Rp) = 1.80% and weighted-profile R factor (Rwp) = 2.69%. Powley-refined triclinic unit cell parameters are: a = 21.12651 Å, b = 20.11224 Å, and c = 3.74284 Å; α = 89.82811°, β = 89.96509°, and γ = 119.62754°.

We need to point out that the PXRD diffraction peaks at ~5.5° for *t*-COF and ~5.2° for *b*-COF can be well-refined when setting the c as 15.90968 Å and 17.25418 Å, respectively. And we found that these two diffraction peak for *t*-COF decreased when the degree of crystallinity increased, and diffraction along 001 facet at ~22.4° become obvious, which indicated that *t*-COF might have two different stacking distances and resulted in two diffraction peaks at ~5.5° and ~22.4°.

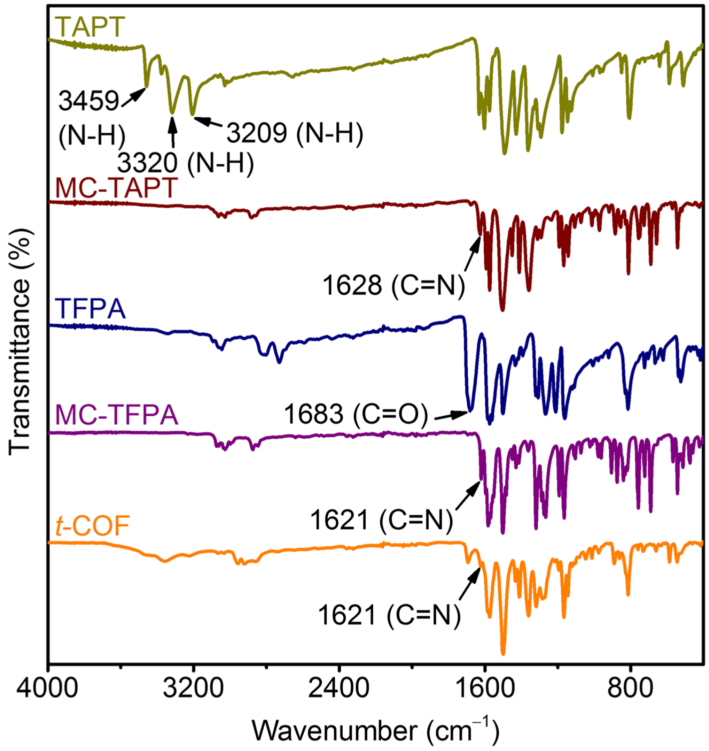

Supplementary Figure 2 | FT-IR spectra of *t*-COF and its corresponding monomers and model compounds.

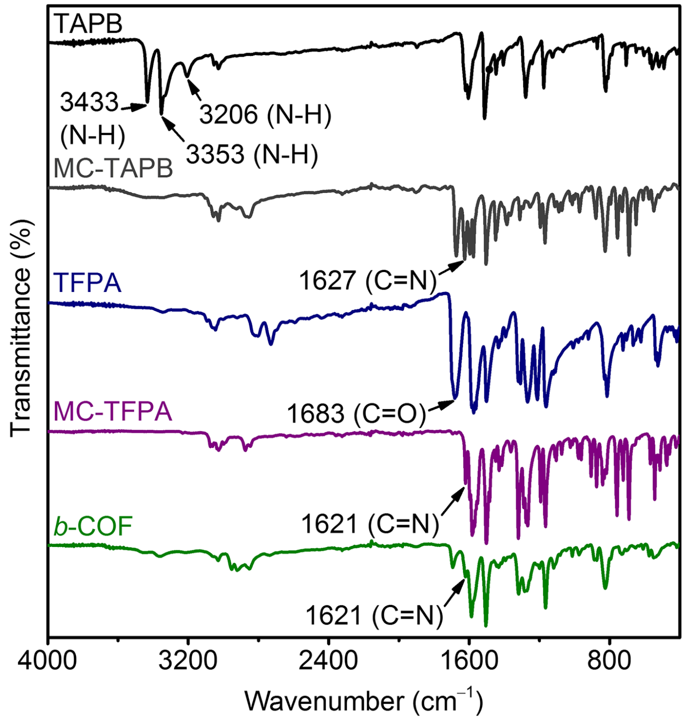

Supplementary Figure 3 | FT-IR spectra of *b*-COF and its corresponding monomers and model compounds.

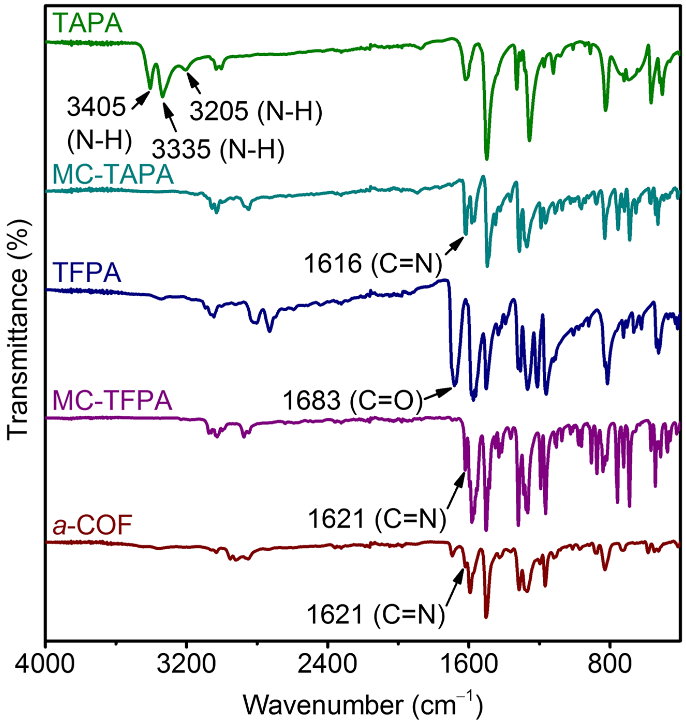

Supplementary Figure 4 | FT-IR spectra of *a*-COF and its corresponding monomers and model compounds.

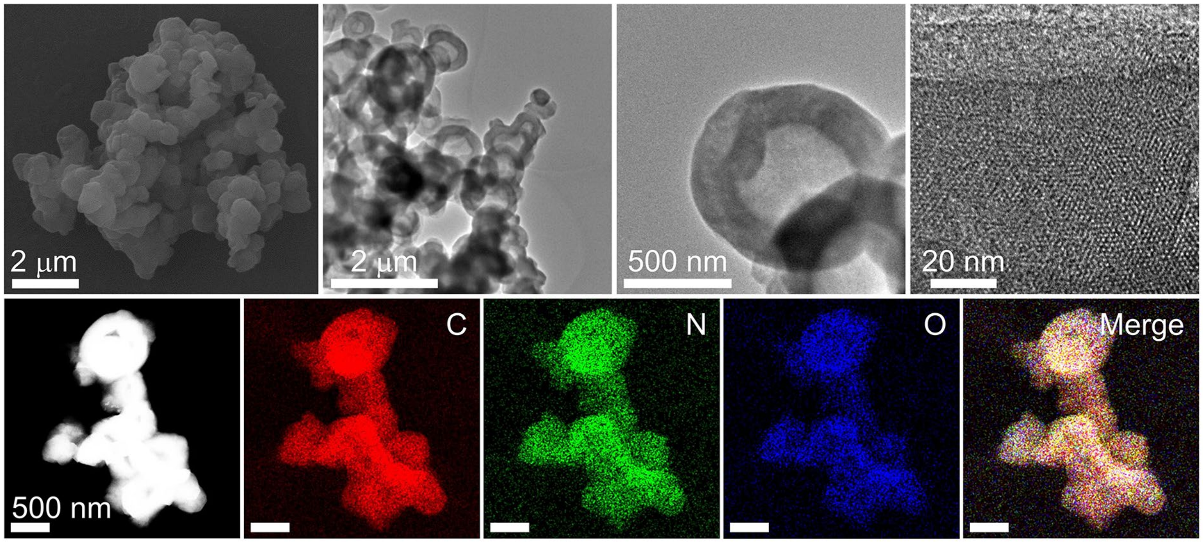

Supplementary Figure 5 | SEM, TEM, and mapping images of *t*-COF.

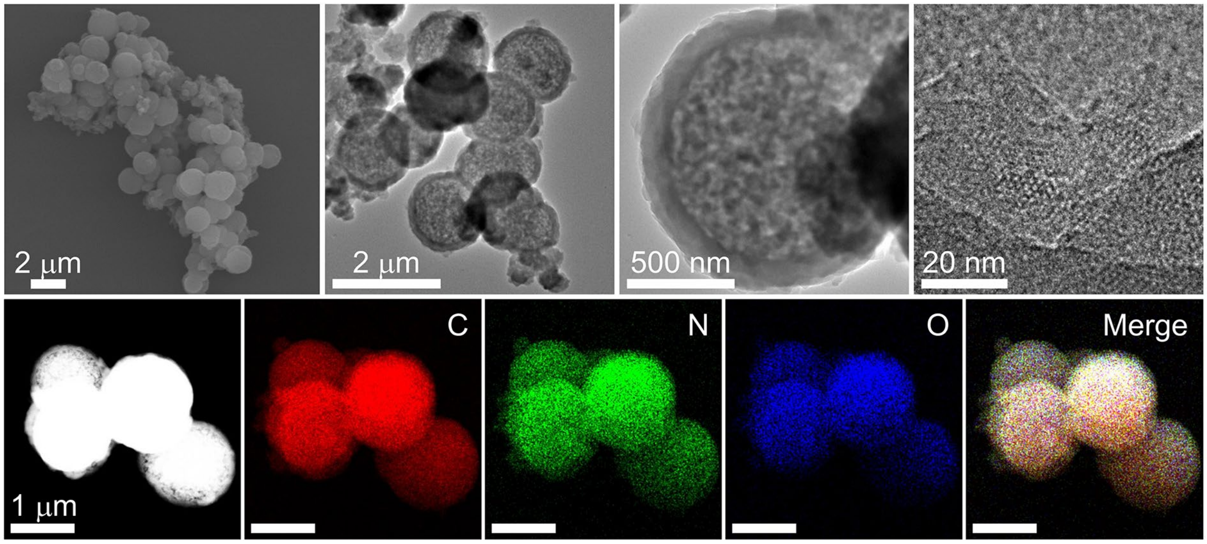

Supplementary Figure 6 | SEM, TEM, and mapping images of *b*-COF.

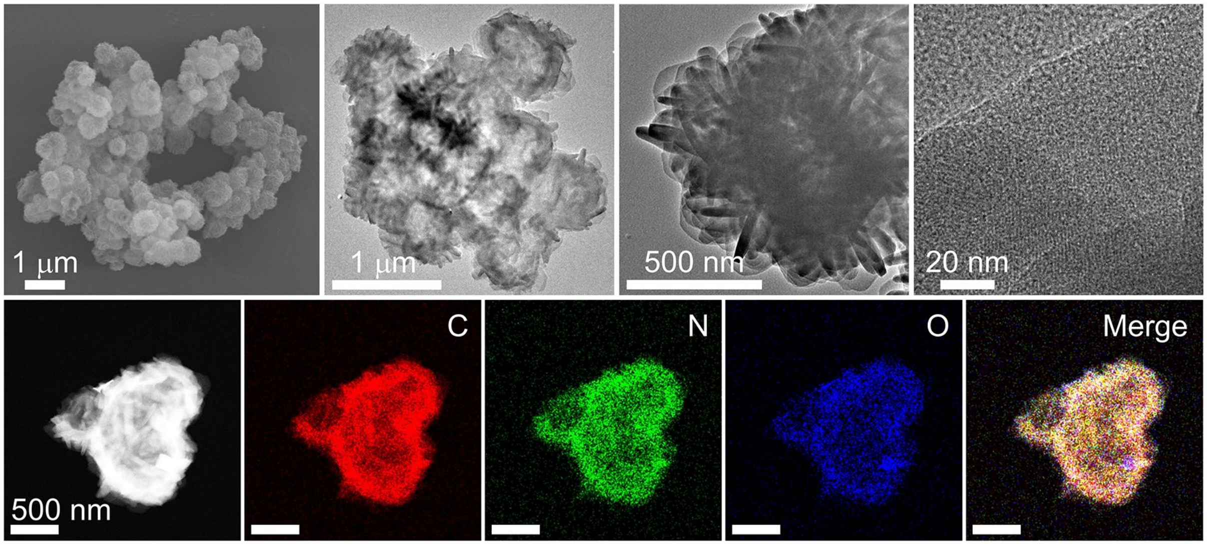

Supplementary Figure 7 | SEM, TEM, and mapping images of *a*-COF.

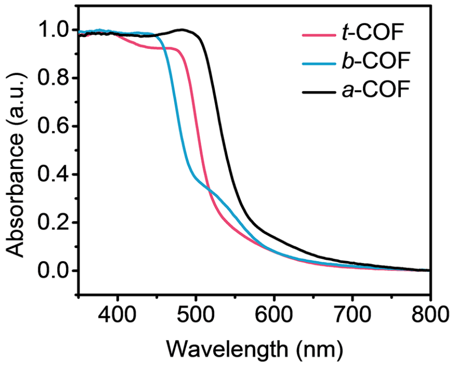

Supplementary Figure 8 | Solid UV-Vis diffuse reflection absorption spectra of *t*-COF, *b*-COF, and *a*-COF.

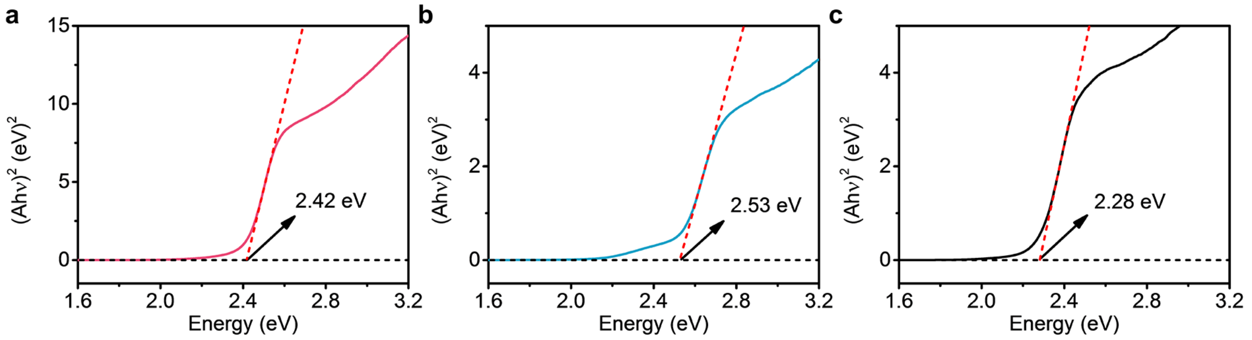

Supplementary Figure 9 | Tauc plots of (**a**) *t*-COF, (**b**) *b*-COF and (**c**) *a*-COF generated from solid-state UV spectra of three COFs.

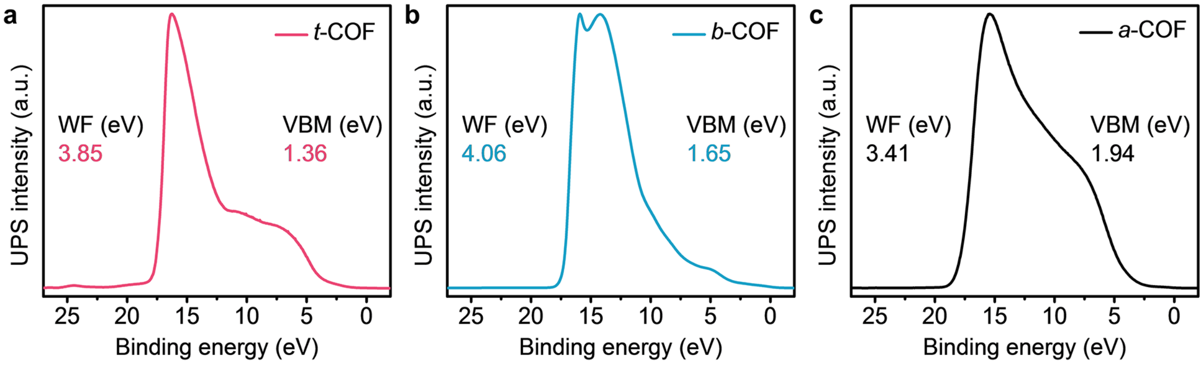

Supplementary Figure 10 | UPS spectra of (**a**) *t*-COF, (**b**) *b*-COF, and (**c**) *a*-COF.

Electronic Structures of 2D *t*-, *b*-, and *a*-COF

Within the HSE06 level, the 2D *a*-COF is calculated to be a semiconductor with a direct bandgap of 2.02 eV. The conduction band minimum (CBM) and valence band maximum (VBM) locate at the Γ point (0, 0, 0). The work function is calculated to be 3.98 eV. The VBM and CBM locate at −4.51 eV and −2.49 eV, respectively. For 2D *b*-COF, the bandgap is 2.61 eV considering the Γ point as CBM and K point (1/3, 1/3, 0) as VBM and the bandgap is 2.66 eV if considering Γ point as CBM and VBM. Here we adopt the Γ point as CBM and VBM considering the direct transition of 2D *b*-COF. The work function is calculated to be 4.85 eV. The VBM and CBM locate at −5.35 eV and −2.69 eV. For 2D *t*-COF, the bandgap is calculated to be 2.63 eV. The CBM and VBM also locate at Γ point (0, 0, 0). The work function is calculated to be 5.09 eV. The VBM and CBM locate at −5.60 eV and −2.97 eV.

Supplementary Table 1 | Experimental and calculated band gaps, VBM, and CBM for 2D *t*-COF, *a*-COF, and *b*-COF.

2D COFs	Experimental band gap (eV)	Calculated band gap (eV)	VBM (eV)	CBM (eV)
t-COF	2.42	2.63	−5.60	−2.97
b-COF	2.53	2.66	−5.35	−2.69
a-COF	2.28	2.02	−4.51	−2.49

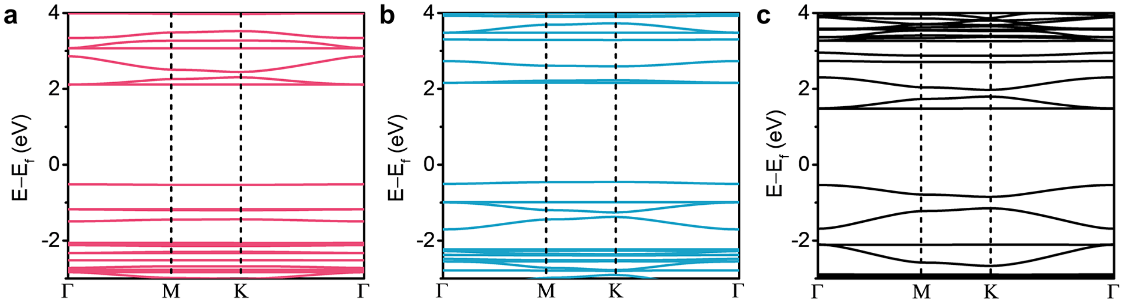

Supplementary Figure 11 | Calculated electronic band structures of (**a**) *t*-COF, (**b**) *b*-COF and (**c**) *a*-COF within HSE06 level.

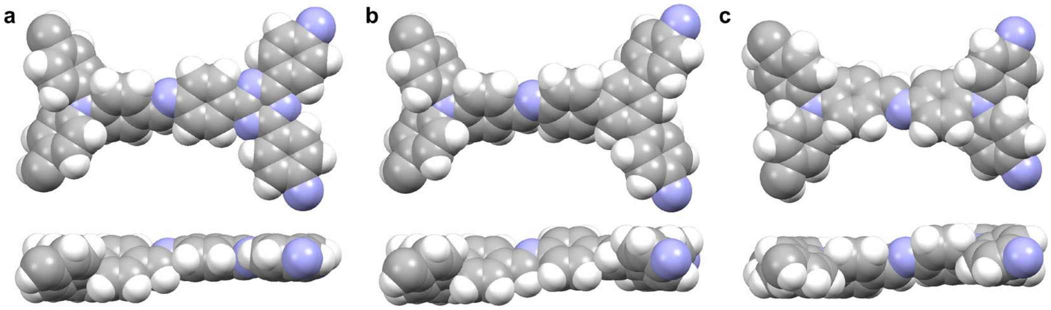

Supplementary Figure 12 | Structure of (**a**) *t*-COF, (**b**) *b*-COF, and (**c**) *a*-COF fragments in top and side views.

ECL Properties of COFs

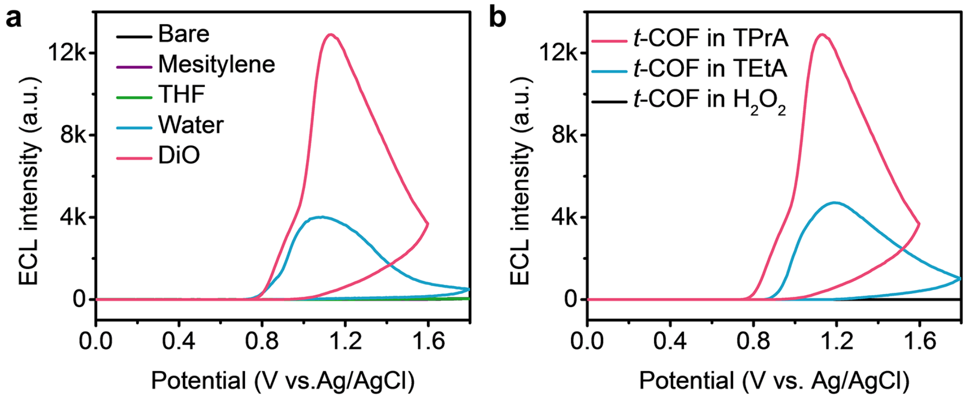

Supplementary Figure 13 | Screen of dispersion solvents and co-reactants. (**a**) ECL curves of *t*-COF modified glassy carbon electrode (GCE) obtained using mesitylene, THF, water, and 1,4-dioxane as the dispersion solvents during the electrode modification process, photomultiplier tube (PMT) voltage = 700 V. (**b**) ECL curves of *t*-COF modified GCEs with 20 mM different co-reactants in PBS (pH = 6.8).

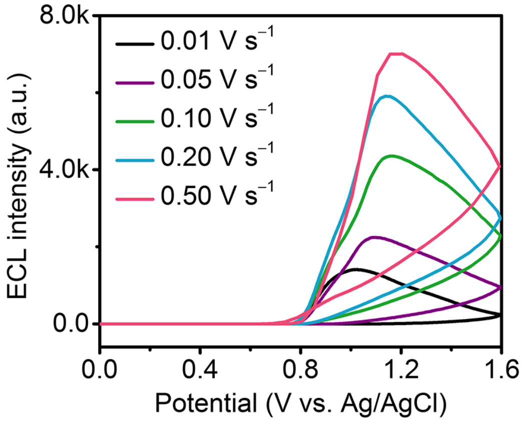

Supplementary Figure 14 | ECL curves of *t*-COF modified GCEs under different scan rates in 0.10 M PBS (pH = 6.8) containing 20 mM TPrA (PMT voltage = 600 V).

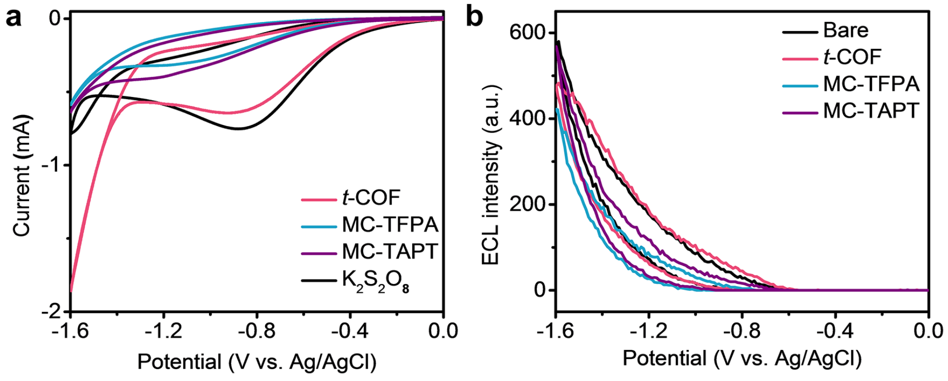

Supplementary Figure 15 | Cathodic ECL behavior of *t*-COF and model compounds. (**a**) CV and (**b**) ECL curves of *t*-COF and corresponding model compounds modified GCEs in 0.10 M PBS (pH = 6.8) containing 0.10 M $K_2S_2O_8$ (PMT voltage = 800 V).

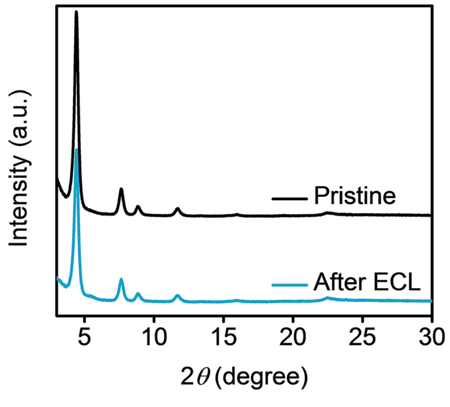

Supplementary Figure 16 | PXRD patterns of *t*-COF before and after ECL test in 0.10 M PBS (pH = 6.8) containing 20 mM TPrA.

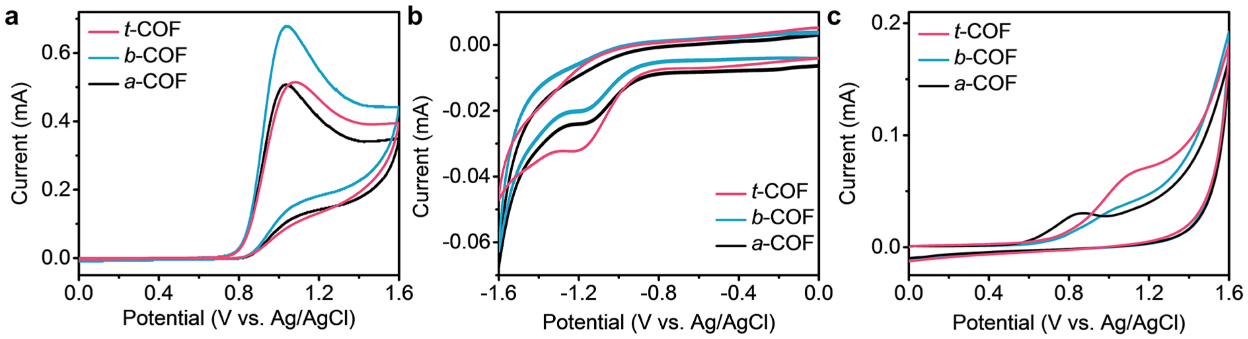

Supplementary Figure 17 | (**a**) CV curves of *a*-COF, *b*-COF, and *t*-COF modified GCEs in 0.10 M PBS containing 20 mM TPrA. (**b**) Cathodic CV curves of *a*-COF, *b*-COF, and *t*-COF modified GCEs in acetonitrile containing 0.10 M $TBAPF_6$ using Ag/Ag^+ as reference electrode, and the potential was calibrated to Ag/AgCl. (**c**) Anodic CV curves of *a*-COF, *b*-COF, and *t*-COF modified GCEs in 0.10 M PBS (pH = 6.8).

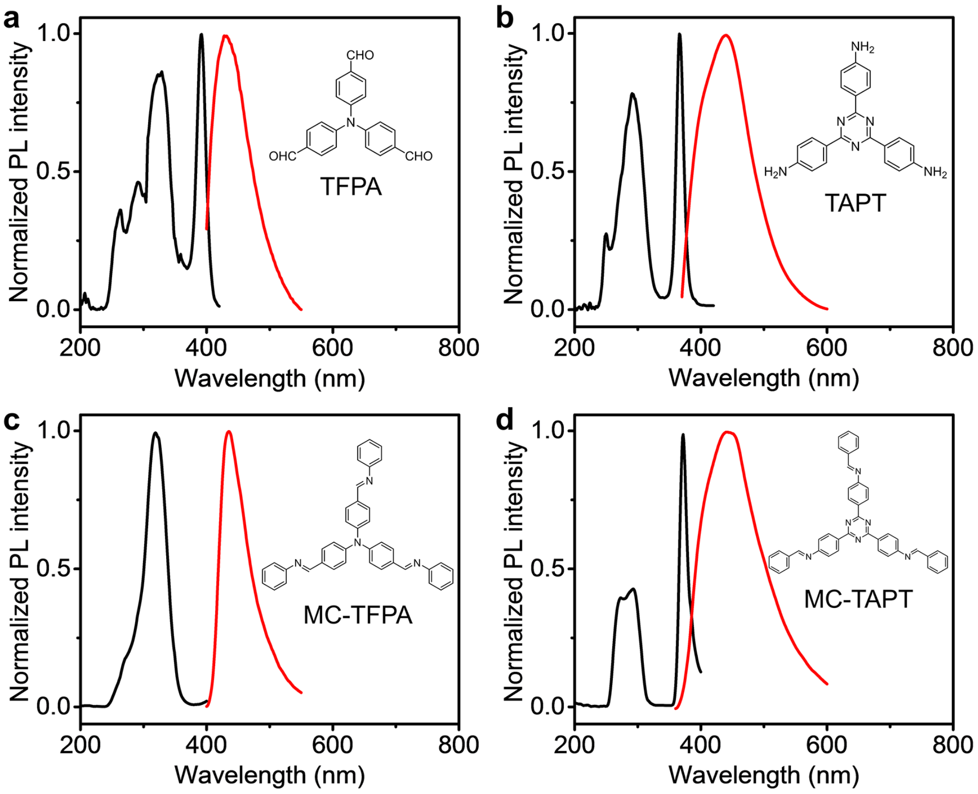

Supplementary Figure 18 | Fluorescence excitation and emission spectra of (**a**) TFPA, (**b**) TAPT, (**c**) MC-TFPA, and (**d**) MC-TAPT.

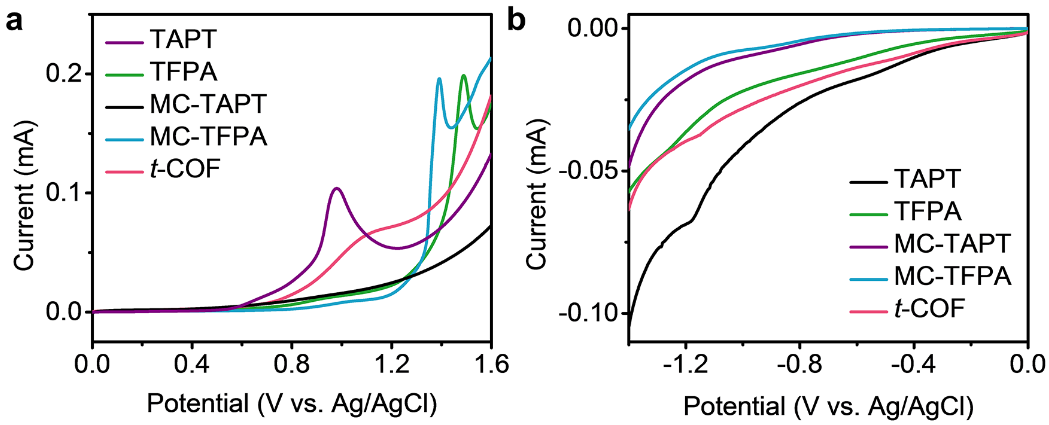

Supplementary Figure 19 | Electrochemical oxidation and reduction behaviors of *t*-COF. (**a**) Anodic and (**b**) cathodic LSV curves of TAPT, TFPA, MC-TAPT, MC-TFPA, and *t*-COF (1.0 mg mL^{-1}) modified GCEs in 0.10 M PBS (pH = 6.8).

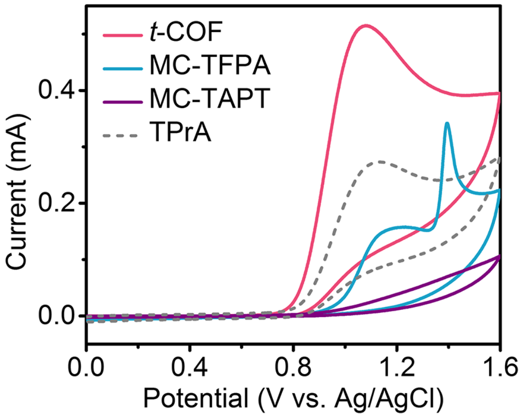

Supplementary Figure 20 | CV curves of *t*-COF, MC-TFPA, and MC-TAPT modified GCEs in 0.10 M PBS (pH = 6.8) containing 20 mM TPrA. Gray dashed line is CV of TPrA at bare GCE.

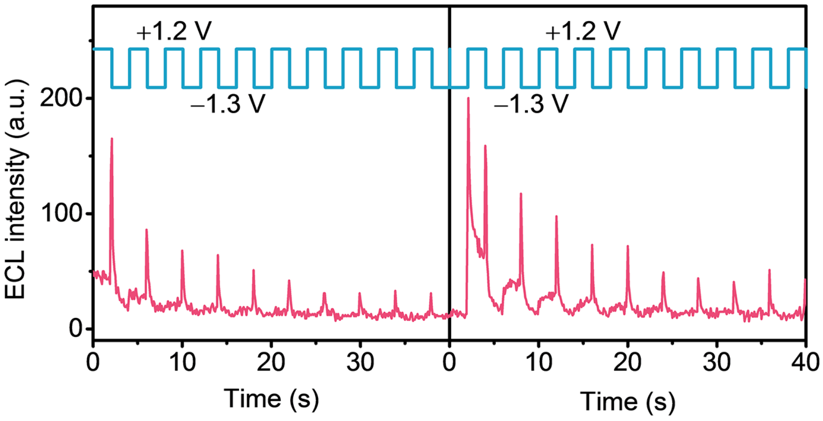

Supplementary Figure 21 | ECL transients of *t*-COF modified GCEs by SP from +1.20 V to −1.30 V (left) and from −1.30 V to +1.20 V (right) in 0.10 M PBS (pH = 6.8) (PMT voltage = 900 V).

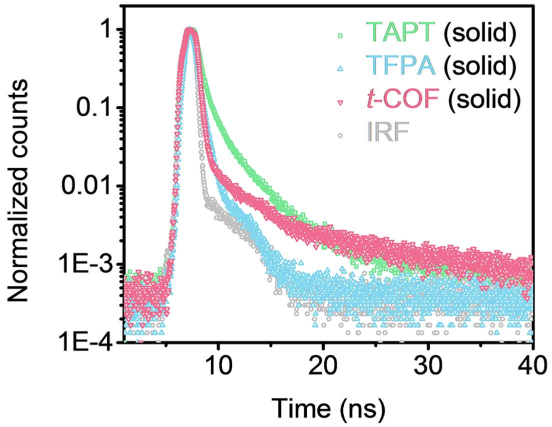

Supplementary Figure 22 | TCSPC traces of solid state TAPT, TFPA, and *t*-COF.

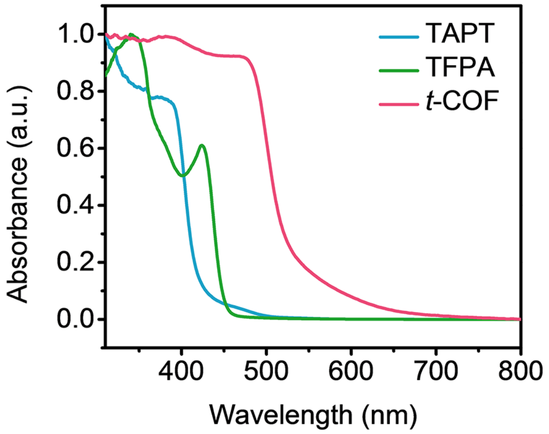

Supplementary Figure 23 | Solid UV-Vis diffuse reflection absorption spectra of TAPT, TFPA, and *t*-COF.

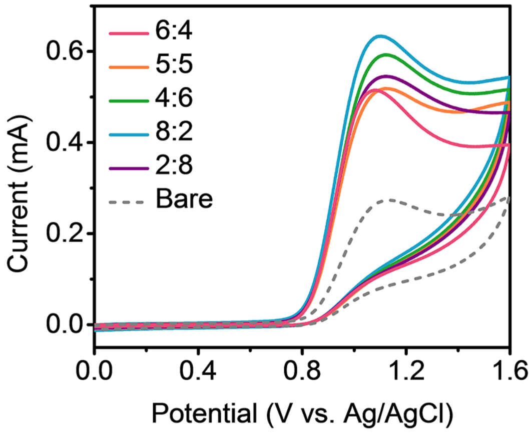

Supplementary Figure 24 | CV curves of *t*-COFs synthesized with different ratios of 1,4-dioxane and mesitylene of 6:4, 5:5, 4:6, 8:2, and 2:8 in 0.10 M PBS (pH = 6.8) containing 20 mM TPrA (PMT voltage = 700 V).

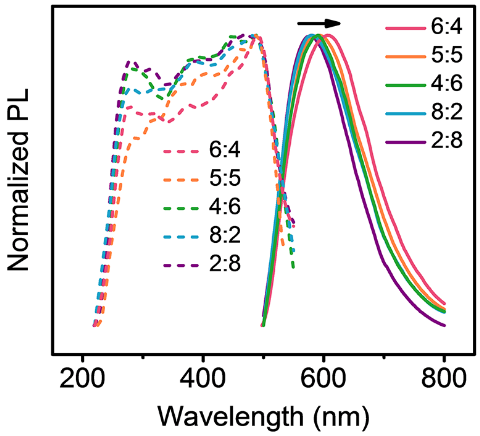

Supplementary Figure 25 | Excitation and emission spectra of *t*-COFs synthesized with different ratios of 1,4-dioxane and mesitylene of 6:4, 5:5, 4:6, 8:2, and 2:8 in 0.10 M PBS (pH = 6.8).

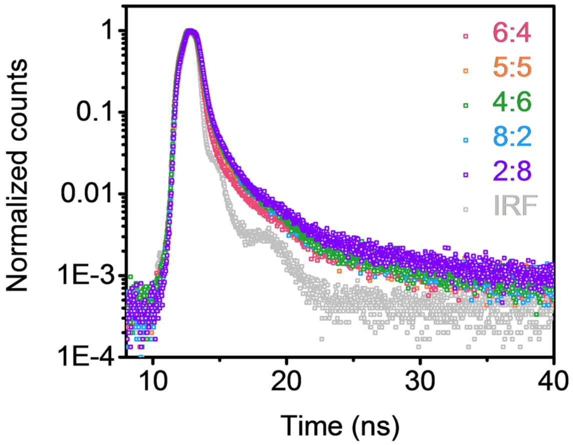

Supplementary Figure 26 | TCSPC traces of *t*-COFs synthesized with different ratios of 1,4-dioxane and mesitylene of 6:4, 5:5, 4:6, 8:2, and 2:8.

Supplementary Table 2 | PL lifetimes of TAPT, TFPA, and *t*-COFs.

	τ_1 (ns)	Rel (%)	τ_2 (ns)	Rel (%)
TAPT	0.90	67	2.18	33
TAPT (solid)	0.75	80	3.70	20
TFPA	1.28	25	2.26	75
TFPA (solid)	0.54	94	3.20	6
t-COF (solid)	0.49	90	9.66	10
t-COF (6:4)	0.54	89	4.37	11
t-COF (5:5)	0.61	89	4.73	11
t-COF (4:6)	0.62	89	4.94	11
t-COF (8:2)	0.63	91	5.50	9
t-COF (2:8)	0.64	89	6.18	11

TAPT and TFPA are dissolved in 1,4-dioxane, and *t*-COFs are dispersed in aqueous environment.

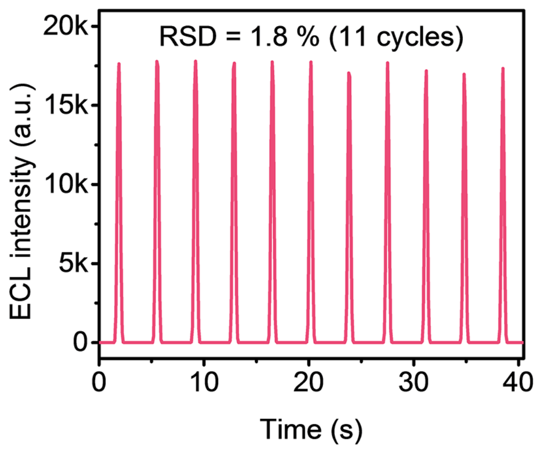

Supplementary Figure 27 | Continuous ECL signals between 0 V and +0.92 V of *t*-COF modified GCE in 0.10 M PBS (pH = 7.6) containing 20 mM TPrA (PMT voltage = 700 V). Scan rate: 0.50 V s^{-1}.

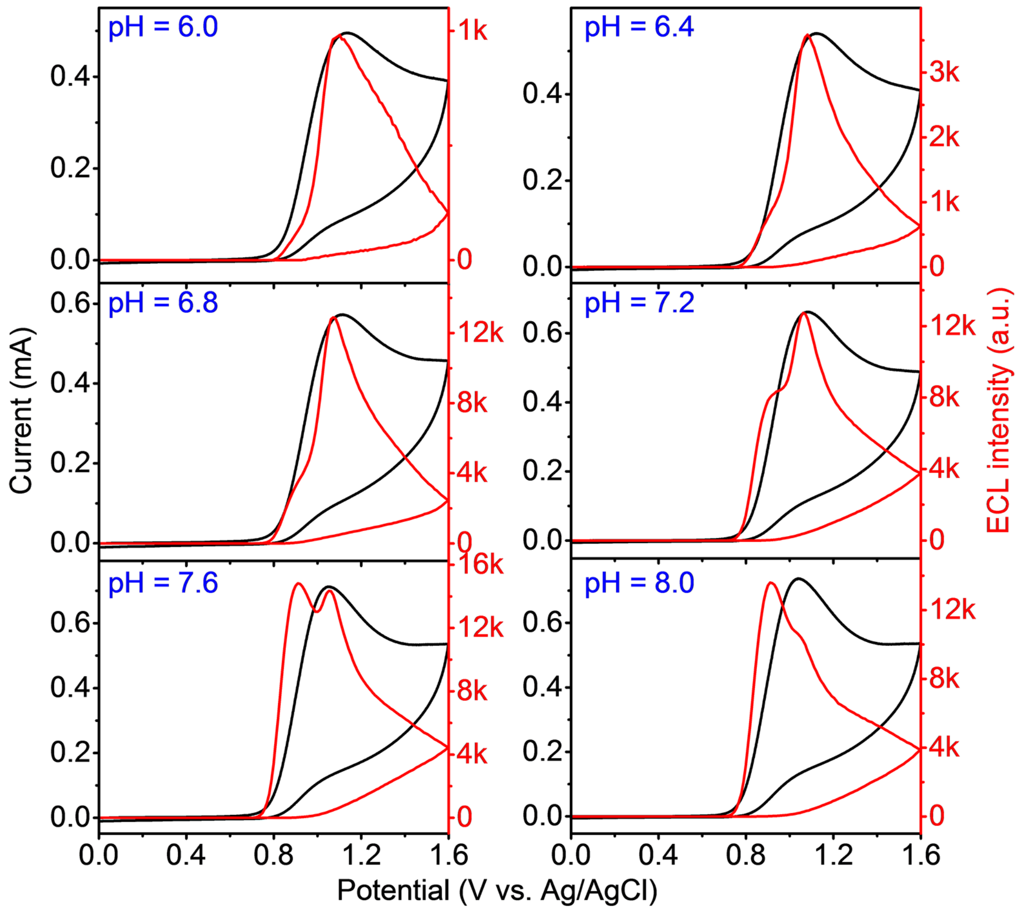

Supplementary Figure 28 | ECL (red) and CV (black) curves of *t*-COF modified GCEs in 0.10 M PBS (pH = 6.0, 6.4, 6.8, 7.2, 7.6, and 8.0) containing 20 mM TPrA (PMT voltage = 700 V).

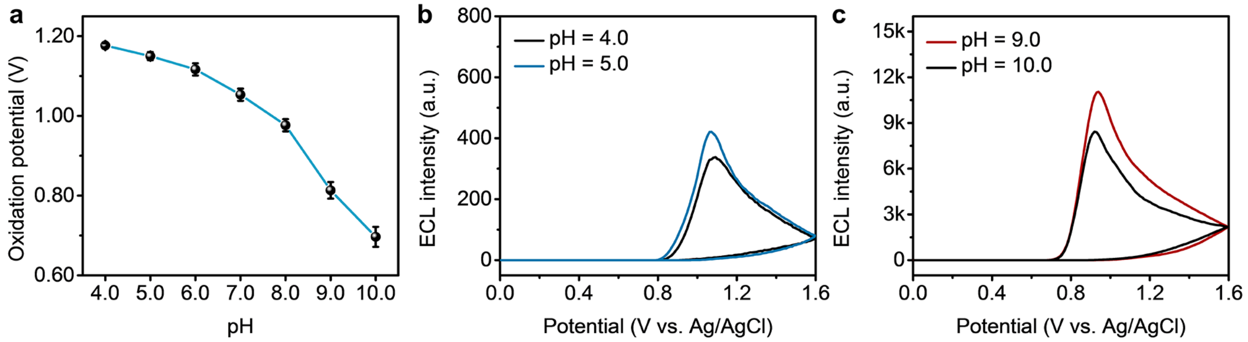

Supplementary Figure 29 | (**a**) Dependence of oxidation potentials vs. Ag/AgCl of TPrA on pHs (the error bars represent the s.d. from triplicate measurements). ECL curves of *t*-COF modified GCEs in (**b**) acidic and (**c**) basic PBS containing 20 mM TPrA (PMT voltage = 700 V).

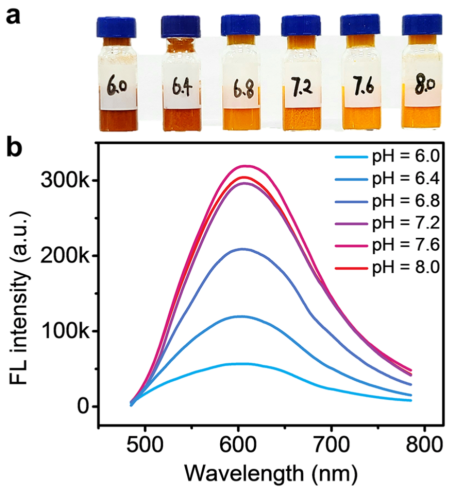

Supplementary Figure 30 | (**a**) Images and (**b**) PL spectra of *t*-COFs in 0.10 M PBS at different pHs.

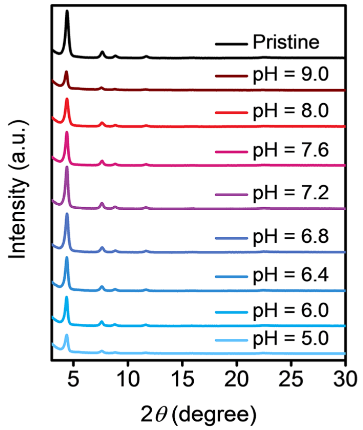

Supplementary Figure 31 | PXRD patterns of *t*-COFs treated with 0.10 M PBS in pH 5.0–9.0 for 12 h.

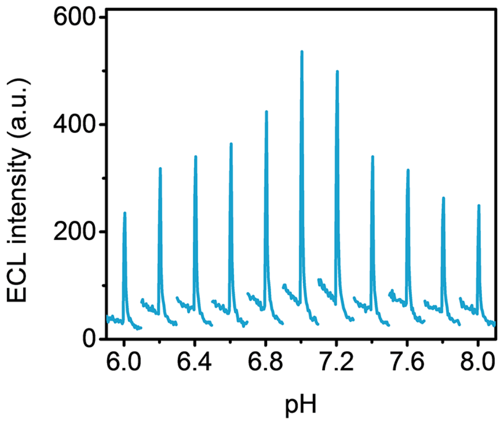

Supplementary Figure 32 | ECL transients of *t*-COF modified GCEs by SP from +1.20 V to −1.30 V in 0.10 M PBS (pH = 6.0–8.0) (PMT voltage = 900 V).

Supplementary Table 3 | Effective mass (m_0) before and after protonation.

m_0	Primitive	Protonated
Electrons	0.23	0.41
Holes	0.04	0.16

The effective mass of primitive 2D *t*-COF is 0.23 m_0 for electron and 0.04 m_0 for hole, which is smaller than the protonated 2D *t*-COF, which is 0.41 m_0 for electron and 0.16 m_0 for hole. According to the deformation theory,[5] the carrier's mobility of pristine 2D *t*-COF is larger than the protonated 2D *t*-COF.

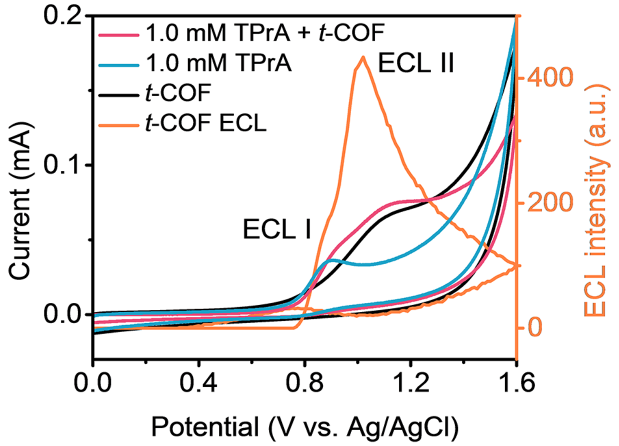

Supplementary Figure 33 | CV curves of 1.0 mM TPrA at bare GCE and *t*-COF modified GCE in 0.10 M PBS (pH = 6.8) without and with 1.0 mM TPrA. ECL curve of *t*-COF modified GCE in 0.10 M PBS (pH = 6.8) containing 1.0 mM TPrA.

IRCT Mediated ECL Generation Mechanism

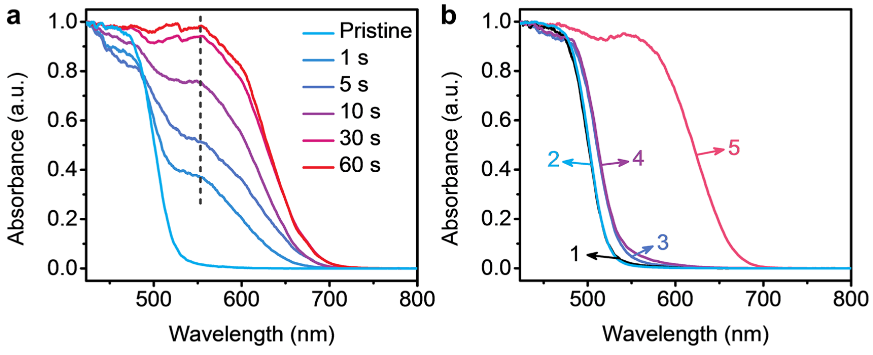

Supplementary Figure 34 | (**a**) UV-Vis spectra of *t*-COF electrolyzed at +1.2 V with different time. (**b**) UV-Vis spectra of *t*-COF before (1) and after electrolyzed at −1.2 V for 10 s (2), electrolyzed at +1.2 V for 10 s and followed at −1.2 V for 10 s (3), LSV scan from 0 V – +1.4 V (5), and followed with scan to −1.4 V and then 0 V (4). Scan rate: 0.10 V s^{-1}.

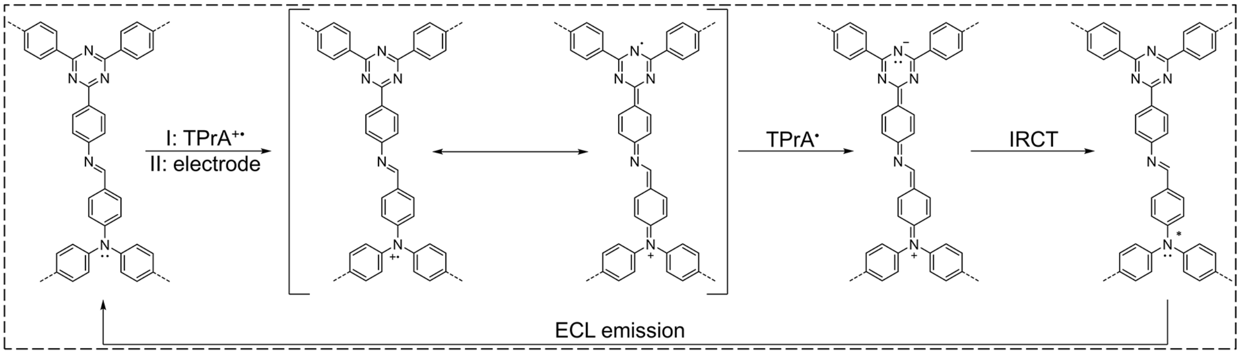

Supplementary Figure 35 | ECL generation pathway of *t*-COF.

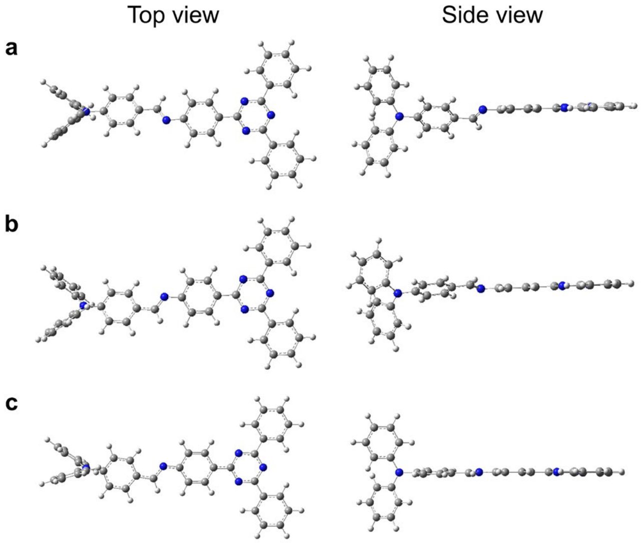

Supplementary Figure 36 | (**a**) Ground state, (**b**) oxidized state, and (**c**) singlet excited state structures of *t*-COF cell fragment in top and side view.

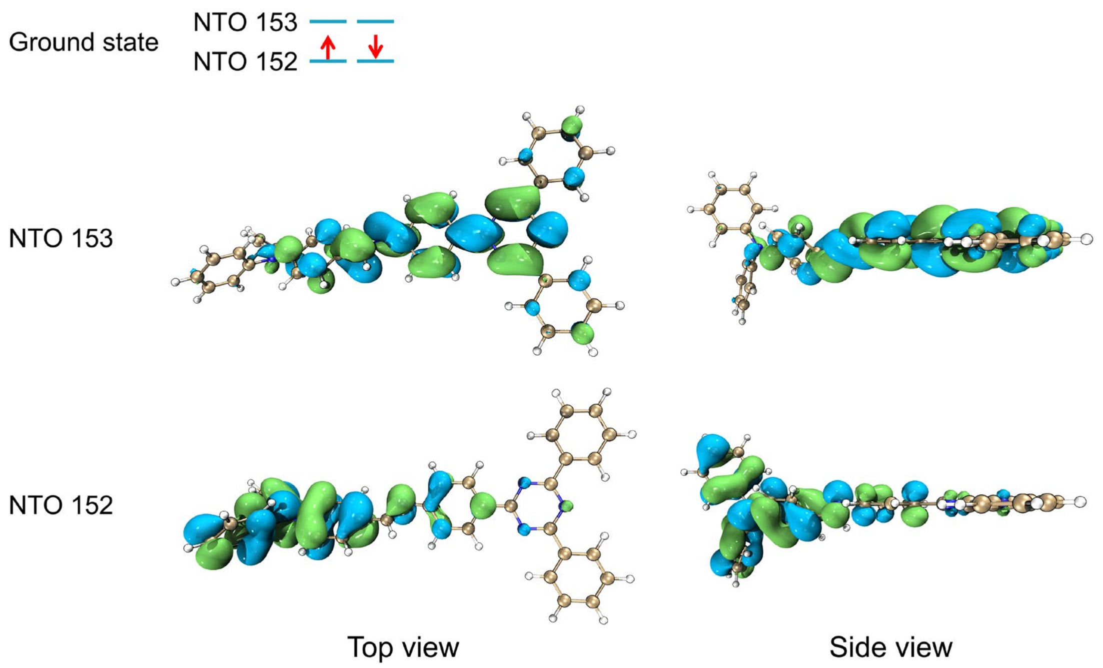

Supplementary Figure 37 | Natural transition orbital (NTO) isodensity surfaces in the ground state of *t*-COF cell fragment in top and side view.

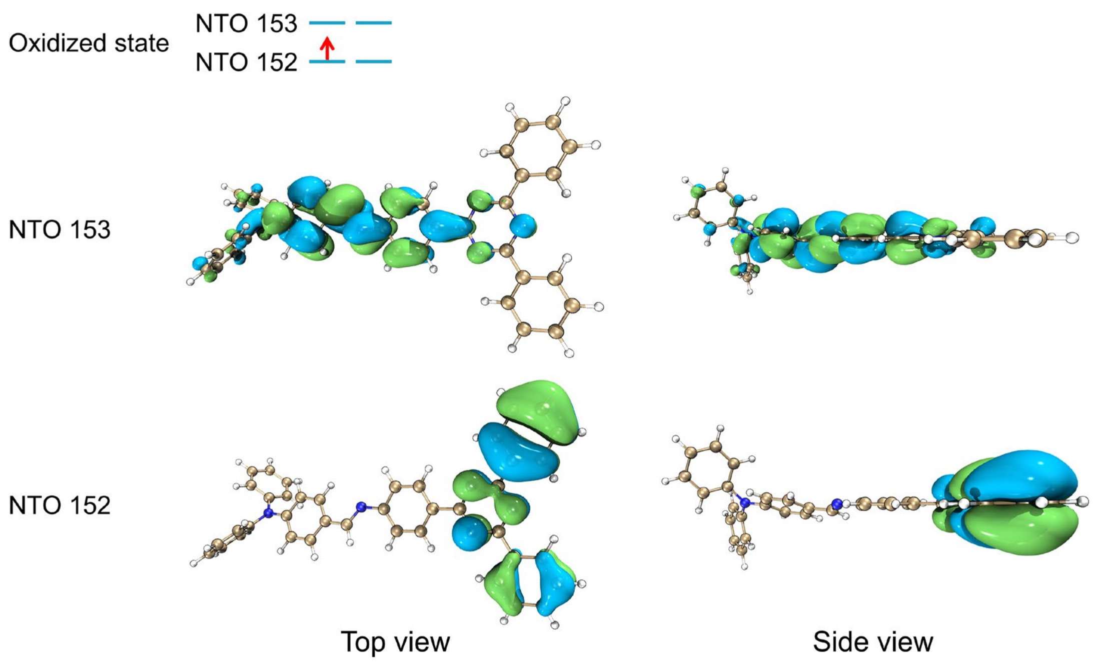

Supplementary Figure 38 | NTO isodensity surfaces in the oxidized state of *t*-COF cell fragment in top and side view.

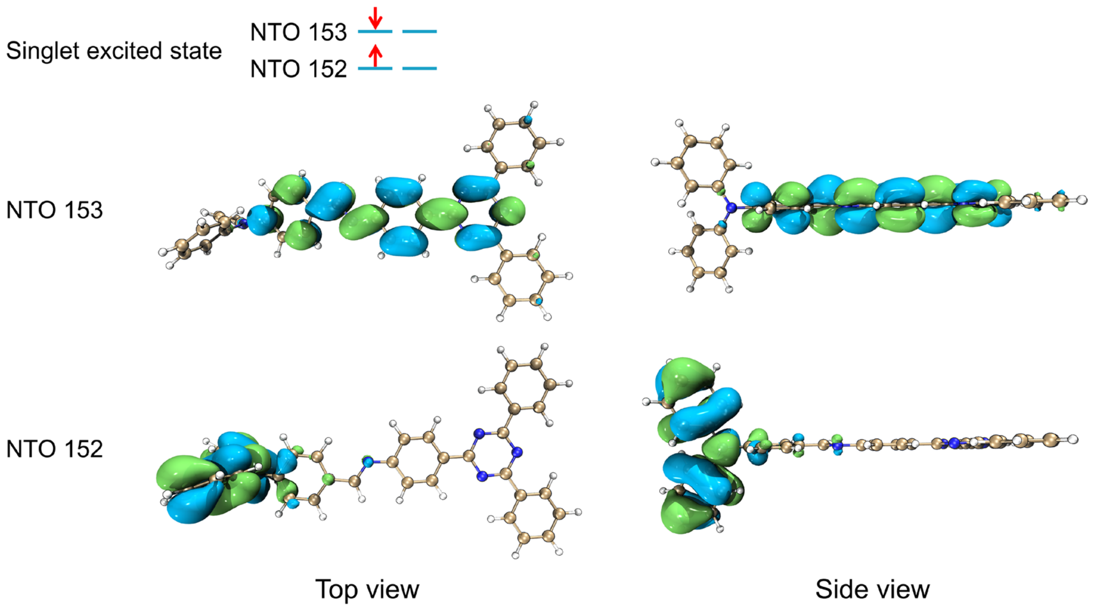

Supplementary Figure 39 | NTO isodensity surfaces in the singlet excited state of *t*-COF cell fragment in top and side view.

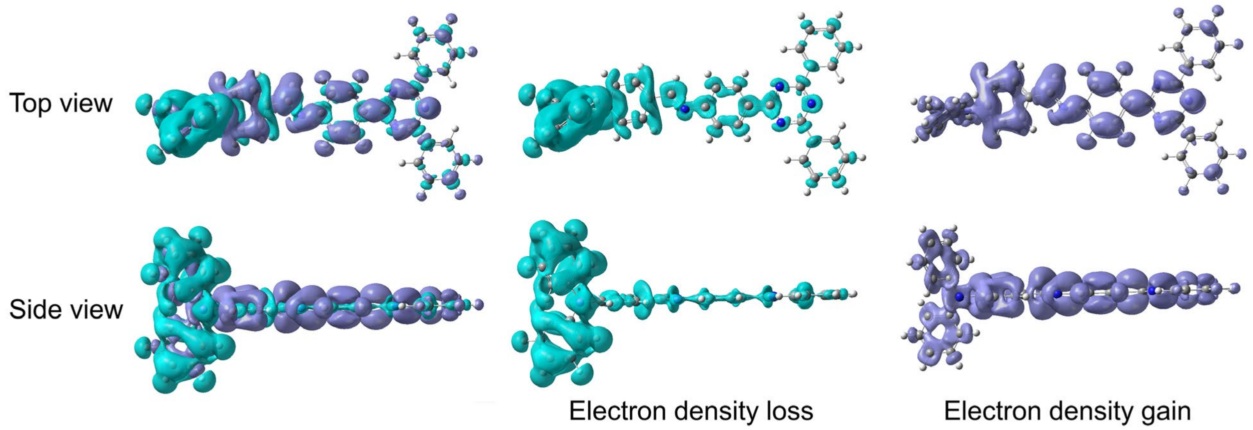

Supplementary Figure 40 | Charge density difference between singlet excited state and ground state of *t*-COF cell fragment. Electron density loss and gain are donated as blue and purple, respectively. When *t*-COF cell fragment was exited, electron migrates from blue to purple areas.

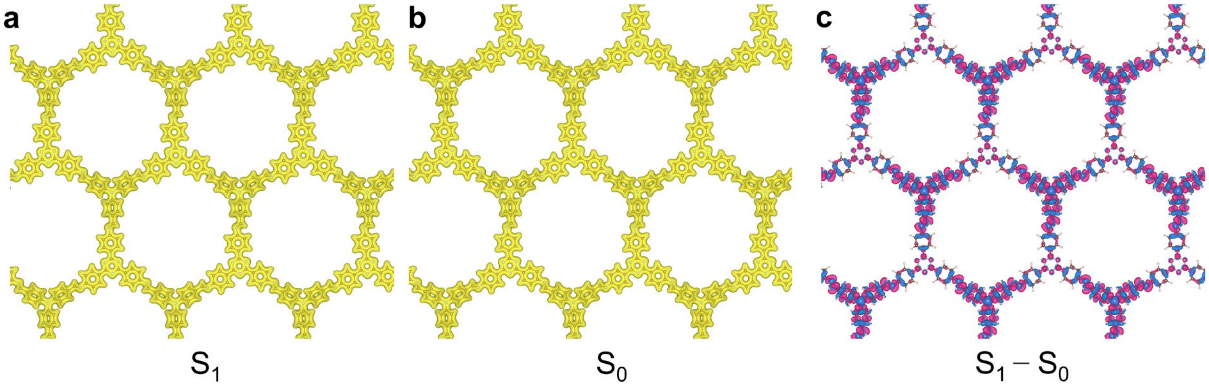

Supplementary Figure 41 | The distribution profiles of charge density of (**a**) 1st excited state, (**b**) ground state, and (**c**) their difference for *t*-COF. Positive (electron density gain) is denoted as blue and negative (electron density loss) is denoted as red.

Structure Stability of *t*-COF after Electron and Hole Doping

Supplementary Table 4 | Lattice parameters (Å) for 2D *t*-COF with $1e^-$ and $1h^+$ doping.

System	a-$1e^-$	a-$1h^+$	a-neutral
t-COF	23.2285	23.1064	23.1879

The whole system of 2D *t*-COF owns total 230 valence electrons. For the electron-doped 2D *t*-COF, the number of valence electrons is set to be 231 and for hole-doped 2D *t*-COF, the number of valence electrons is 229. A homogeneous background charge is added to neutralize the whole system. Based on the above method, the electron- and hole-doped *t*-COF was optimized and the framework of structural configurations remains unchanged except the lattice parameters of electron- and hole-doped *t*-COF are a = 23.2285 and 23.1064 Å, respectively, which is slightly larger and smaller than the pristine *t*-COF.

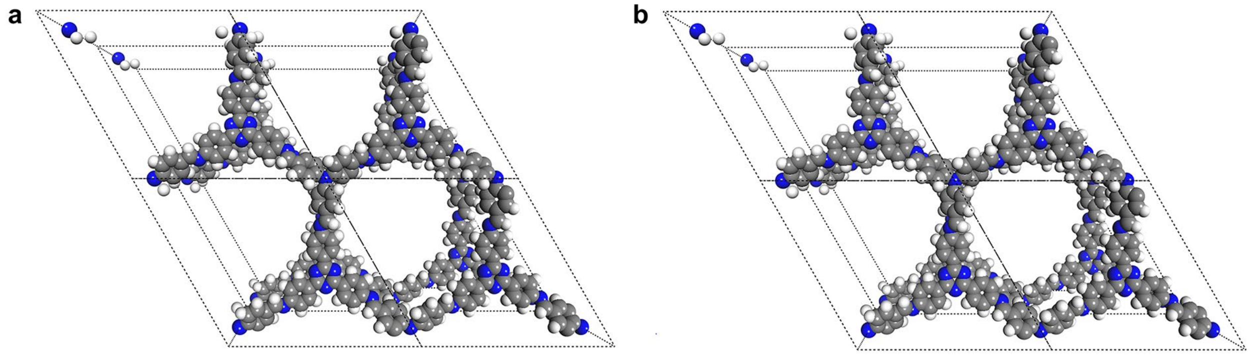

Supplementary Figure 42 | (**a**) Electrons and (**b**) holes doped 2D *t*-COF structures.

Supplementary Table 5 | Fractional atomic coordinates for bulk *t*-COF.

t-COF			
Formula: $C_{42}N_7H_{27}$<br>Weight: 629.722 g mol^{-1}<br>Crystal system: triclinic<br>Space-group: P1 (1)<br>a = 23.11142 Å; b = 23.22254 Å; c = 15.90968 Å<br>α = 89.82975°; β = 89.99319°; γ = 119.95484°			
Atom	*x*	*y*	*z*
C1	0.07042	0.03734	0.47864
C2	0.96529	0.03185	0.47849
C3	0.96999	0.92752	0.47689
C4	0.10379	0.09526	0.52746
C5	0.90708	0.00715	0.52748
C6	0.99485	0.89427	0.52588
C7	0.17309	0.13165	0.52747
C8	0.87048	0.03975	0.52751
C9	0.96214	0.8253	0.52609
C10	0.21083	0.11165	0.47891
C11	0.89052	0.09727	0.47881
C12	0.90435	0.78777	0.47758
C13	0.17687	0.05338	0.43028
C14	0.94909	0.12171	0.43003
C15	0.87975	0.82161	0.42879
C16	0.10782	0.01667	0.43015
C17	0.98602	0.0897	0.42987
C18	0.91186	0.89033	0.42845
C19	0.38921	0.17748	0.43241
C20	0.82425	0.20895	0.43228
C21	0.79224	0.61027	0.43156
C22	0.4288	0.14735	0.42356
C23	0.85447	0.27844	0.42326
C24	0.72243	0.57091	0.42276
C25	0.4979	0.18564	0.42301
C26	0.81595	0.30891	0.42274
C27	0.69186	0.50214	0.42241
C28	0.52961	0.25531	0.42902
C29	0.74595	0.27085	0.42896
C30	0.73015	0.47054	0.4286
C31	0.49015	0.28564	0.43471
C32	0.71553	0.2013	0.43482
C33	0.80003	0.50975	0.43425
C34	0.42101	0.24748	0.43638
C35	0.75392	0.17065	0.43646
C36	0.83078	0.57857	0.43572
C37	0.60307	0.29599	0.42862
C38	0.70503	0.30329	0.42859
C39	0.6976	0.39742	0.42843
C40	0.28337	0.15106	0.4796
C41	0.85088	0.13005	0.47954
C42	0.87146	0.71558	0.47848
N1	0.32012	0.13644	0.4336
N2	0.86553	0.18121	0.43342
N3	0.82006	0.67903	0.43254

N4	0.73711	0.37015	0.42849
N5	0.63042	0.36252	0.42852
N6	0.63818	0.26402	0.42869
N7	0	0	0.478
H1	0.07486	0.11103	0.56533
H2	0.89128	0.96116	0.56367
H3	0.0367	0.92394	0.56388
H4	0.19873	0.17629	0.56616
H5	0.82562	0.02065	0.56633
H6	0.98136	0.79977	0.56491
H7	0.20632	0.03827	0.39234
H8	0.96423	0.1661	0.39198
H9	0.83516	0.79233	0.39088
H10	0.08173	0.97021	0.39053
H11	0.02835	0.10958	0.39208
H12	0.89277	0.91632	0.39049
H13	0.40378	0.09336	0.41823
H14	0.90872	0.30748	0.41776
H15	0.69321	0.59585	0.41729
H16	0.52874	0.16251	0.41745
H17	0.83916	0.36271	0.41704
H18	0.6378	0.47148	0.41688
H19	0.51491	0.33971	0.43771
H20	0.66121	0.17191	0.43798
H21	0.82959	0.48508	0.43739
H22	0.39083	0.27157	0.43902
H23	0.72975	0.11654	0.43924
H24	0.88514	0.60857	0.43833
H25	0.89262	0.69367	0.52206
H26	0.30536	0.194	0.52305
H27	0.80773	0.10903	0.52312

Supplementary Table 6 | Fractional atomic coordinates for bulk *b*-COF.

b-COF			
Formula: $C_{45}N_4H_{30}$<br>Weight: 626.759 g mol^{-1}<br>Crystal system: triclinic<br>Space-group: P1 (1)<br>a = 23.46845 Å; b = 23.43171 Å; c = 17.25418 Å<br>α = 90.00000°; β = 90.00000°; γ = 119.99866°			
Atom	*x*	*y*	*z*
C1	0.06936	0.03747	0.452
C2	0.9617	0.03197	0.452
C3	0.96717	0.9312	0.452
C4	0.10118	0.09341	0.49969
C5	0.90585	0.00789	0.49969
C6	0.99121	0.89933	0.49969
C7	0.16945	0.12932	0.50224
C8	0.87	0.04037	0.50224
C9	0.95878	0.83095	0.50224
C10	0.20765	0.11132	0.45658
C11	0.88796	0.09662	0.45658
C12	0.90262	0.7927	0.45658
C13	0.17521	0.05535	0.40848

C14	0.94384	0.1201	0.40848
C15	0.87918	0.82518	0.40848
C16	0.1072	0.01868	0.40662
C17	0.98046	0.08866	0.40662
C18	0.91057	0.89331	0.40662
C19	0.38455	0.18249	0.41218
C20	0.81691	0.20264	0.41218
C21	0.79677	0.61552	0.41218
C22	0.42087	0.16005	0.36995
C23	0.83931	0.26145	0.36995
C24	0.73806	0.57914	0.36995
C25	0.48916	0.19646	0.36711
C26	0.80295	0.29344	0.36711
C27	0.70611	0.51075	0.36711
C28	0.52411	0.25723	0.40588
C29	0.74227	0.26768	0.40588
C30	0.73184	0.47574	0.40588
C31	0.48763	0.28001	0.44723
C32	0.71953	0.20835	0.44723
C33	0.79108	0.51227	0.44723
C34	0.41945	0.24388	0.45017
C35	0.75561	0.1762	0.45017
C36	0.82317	0.58056	0.45017
C37	0.59665	0.29622	0.40376
C38	0.70335	0.30135	0.40376
C39	0.69822	0.40308	0.40376
C40	0.73456	0.37036	0.40357
C41	0.62932	0.36533	0.40357
C42	0.63435	0.26496	0.40357
C43	0.27903	0.15079	0.45948
C44	0.84856	0.12866	0.45948
C45	0.87064	0.7212	0.45948
N1	0.31608	0.14247	0.41101
N2	0.85686	0.17407	0.41101
N3	0.8253	0.68409	0.41101
N4	0	0	0.44983
H1	0.07184	0.10774	0.53496
H2	0.89154	0.96488	0.53496
H3	0.03574	0.92874	0.53496
H4	0.19396	0.17223	0.54024
H5	0.82715	0.022	0.54024
H6	0.97712	0.8064	0.54024
H7	0.205	0.04193	0.37267
H8	0.95723	0.16336	0.37267
H9	0.83599	0.79535	0.37267
H10	0.08219	0.97582	0.36988
H11	0.02484	0.10719	0.36988
H12	0.89209	0.91835	0.36988
H13	0.39374	0.11341	0.33896
H14	0.88588	0.28092	0.33896
H15	0.71861	0.60631	0.33896
H16	0.51588	0.1784	0.33262
H17	0.82098	0.33826	0.33262
H18	0.66136	0.48398	0.33262
H19	0.51352	0.32662	0.47858

H20	0.67299	0.18767	0.47858
H21	0.81172	0.48635	0.47858
H22	0.39319	0.26389	0.48195
H23	0.73563	0.12989	0.48195
H24	0.86941	0.60686	0.48195
H25	0.78817	0.39916	0.40374
H26	0.60057	0.39023	0.40374
H27	0.60948	0.21126	0.40374
H28	0.29892	0.18826	0.50609
H29	0.81114	0.1111	0.50609
H30	0.88816	0.70127	0.50609

Supplementary Table 7 | Fractional atomic coordinates for bulk *a*-COF.

a-COF			
Formula: $C_{39}N_5H_{27}$<br>Weight: 565.676 g mol^{-1}<br>Crystal system: triclinic<br>Space-group: P1 (1)<br>a = 21.12651 Å; b = 20.11224 Å; c = 3.74284 Å<br>α = 89.82811°; β = 89.96509°; γ = 119.62754°			
Atom	*x*	*y*	*z*
C1	0.36689	0.64915	0.8566
C2	0.36826	0.77221	0.85461
C3	0.25154	0.65151	0.85696
C4	0.43476	0.67881	0.00976
C5	0.33962	0.8138	0.00791
C6	0.21231	0.58025	0.01212
C7	0.47132	0.63771	0.02961
C8	0.37833	0.89285	0.02581
C9	0.13704	0.5423	0.03229
C10	0.44107	0.56361	0.90274
C11	0.44883	0.93474	0.89673
C12	0.09678	0.57451	0.90375
C13	0.37393	0.53471	0.72516
C14	0.47675	0.89315	0.71903
C15	0.13601	0.645	0.72421
C16	0.33784	0.57635	0.70096
C17	0.43753	0.81406	0.69678
C18	0.21132	0.68245	0.69968
C19	0.6249	0.38845	0.11055
C20	0.61359	0.26065	0.11265
C21	0.73489	0.37566	0.11038
C22	0.55725	0.36412	0.96134
C23	0.63717	0.214	0.96348
C24	0.77896	0.44664	0.95924
C25	0.52528	0.40972	0.956
C26	0.59416	0.13529	0.96019
C27	0.85393	0.47976	0.95374
C28	0.55941	0.48218	0.10053
C29	0.52518	0.09908	0.10683
C30	0.88878	0.4435	0.10002
C31	0.62659	0.50629	0.26993
C32	0.50182	0.14547	0.27617
C33	0.84497	0.37302	0.27133
C34	0.65877	0.46062	0.27487

C35	0.54489	0.22447	0.27907
C36	0.76972	0.33967	0.27644
C37	0.52766	0.53177	0.09289
C38	0.47839	0.01664	0.10137
C39	0.96733	0.47635	0.09227
N1	0.4715	0.51596	0.91728
N2	0.4938	1.01387	0.90917
N3	0.02139	0.54302	0.91839
N4	0.6578	0.34159	0.11202
N5	0.32889	0.69096	0.8448
H1	0.45891	0.73502	0.13165
H2	0.28614	0.78329	0.13146
H3	0.24164	0.55456	0.1353
H4	0.52305	0.66307	0.17156
H5	0.3539	0.92182	0.16797
H6	0.10974	0.48797	0.1758
H7	0.35133	0.47863	0.60054
H8	0.53011	0.92517	0.59273
H9	0.10525	0.66906	0.59832
H10	0.28627	0.55215	0.56041
H11	0.46086	0.78411	0.55607
H12	0.23956	0.7366	0.55759
H13	0.52998	0.30874	0.83144
H14	0.6899	0.24042	0.83199
H15	0.7535	0.47559	0.82798
H16	0.47386	0.39096	0.81751
H17	0.61234	0.10008	0.82172
H18	0.88719	0.53371	0.81379
H19	0.65341	0.56142	0.40604
H20	0.44933	0.11882	0.41388
H21	0.87063	0.34453	0.40879
H22	0.71064	0.48084	0.40844
H23	0.52532	0.25869	0.41266
H24	0.73741	0.28524	0.4115
H25	0.55491	0.58304	0.26373
H26	0.42955	1.03459	0.25688
H27	0.00222	0.45523	0.26769

Supplementary Table 8 | Fractional atomic coordinates for crystal structure model of protonated 2D *t*-COF.

Protonated 2D *t*-COF			
Formula: $C_{42}N_7H_{28}$<br>Weight: 630.73 g mol^{-1}<br>Crystal system: triclinic<br>Space-group: P1 (1)<br>a = 23.19291 Å; b = 23.23387 Å; c = 15.00000 Å<br>α = 90.00000°; β = 90.00000°; γ = 119.88670°			
Atom	*x*	*y*	*z*
C1	0.07024	0.03844	0.50743
C2	0.10363	0.09618	0.55965
C3	0.17261	0.13229	0.55971
C4	0.21014	0.11226	0.50789
C5	0.17612	0.05415	0.45589
C6	0.10738	0.01764	0.45587
C7	0.38768	0.17749	0.45892

C8	0.42736	0.14757	0.44971
C9	0.49614	0.18592	0.44839
C10	0.52756	0.25557	0.45379
C11	0.48801	0.28566	0.46014
C12	0.41921	0.24745	0.46271
C13	0.60064	0.29643	0.45277
C14	0.28228	0.15127	0.5089
C15	0.96257	0.03352	0.50864
C16	0.90448	0.00871	0.56044
C17	0.86782	0.0411	0.56041
C18	0.8874	0.09837	0.50842
C19	0.94573	0.12277	0.45652
C20	0.98286	0.09114	0.4568
C21	0.82162	0.21021	0.45947
C22	0.85162	0.27966	0.45016
C23	0.81323	0.31012	0.4493
C24	0.74347	0.27208	0.45515
C25	0.7133	0.20261	0.46139
C26	0.75156	0.17198	0.46363
C27	0.70253	0.30429	0.45412
C28	0.84798	0.13115	0.50923
C29	0.96804	0.93121	0.50822
C30	0.99454	0.89812	0.5581
C31	0.96402	0.82991	0.55746
C32	0.90477	0.79025	0.50735
C33	0.87877	0.82463	0.45734
C34	0.90965	0.89309	0.45794
C35	0.78671	0.60991	0.46595
C36	0.71763	0.57066	0.45119
C37	0.68804	0.50227	0.44614
C38	0.72623	0.4707	0.45496
C39	0.79542	0.51034	0.46635
C40	0.8256	0.57885	0.47184
C41	0.69442	0.39799	0.45344
C42	0.87546	0.72089	0.51006
N1	0.31897	0.13637	0.46049
N2	0.73415	0.37104	0.45453
N3	0.86282	0.18261	0.46081
N4	0.62747	0.36283	0.45207
N5	0.81462	0.67804	0.47542
N6	0.63593	0.26475	0.45355
N7	0.00013	0.00098	0.5077
H1	0.07494	0.11183	0.60031
H2	0.19832	0.17668	0.60121
H3	0.20525	0.0389	0.41541
H4	0.08126	0.97255	0.41602
H5	0.40259	0.09361	0.44475
H6	0.52691	0.16279	0.44269
H7	0.5125	0.33964	0.46305
H8	0.38901	0.27142	0.46571
H9	0.90087	0.69841	0.54392
H10	0.78464	0.69685	0.45921
H11	0.88905	0.9644	0.60082
H12	0.82333	0.02229	0.60191
H13	0.96069	0.16696	0.41596

H14	0.02796	0.11011	0.4168
H15	0.90565	0.30867	0.44483
H16	0.83637	0.36387	0.44346
H17	0.65921	0.17332	0.46434
H18	0.7275	0.11794	0.46668
H19	0.3042	0.1943	0.55489
H20	0.03942	0.92723	0.5975
H21	0.98471	0.80529	0.59753
H22	0.83541	0.7973	0.41437
H23	0.88974	0.91842	0.41793
H24	0.6873	0.59445	0.4455
H25	0.63458	0.47169	0.43617
H26	0.82506	0.48594	0.47087
H27	0.87941	0.60849	0.47918
H28	0.80488	0.11004	0.55519

^{13}C NMR spectroscopy

MC-TAPT 150 MHz, $CDCl_3$

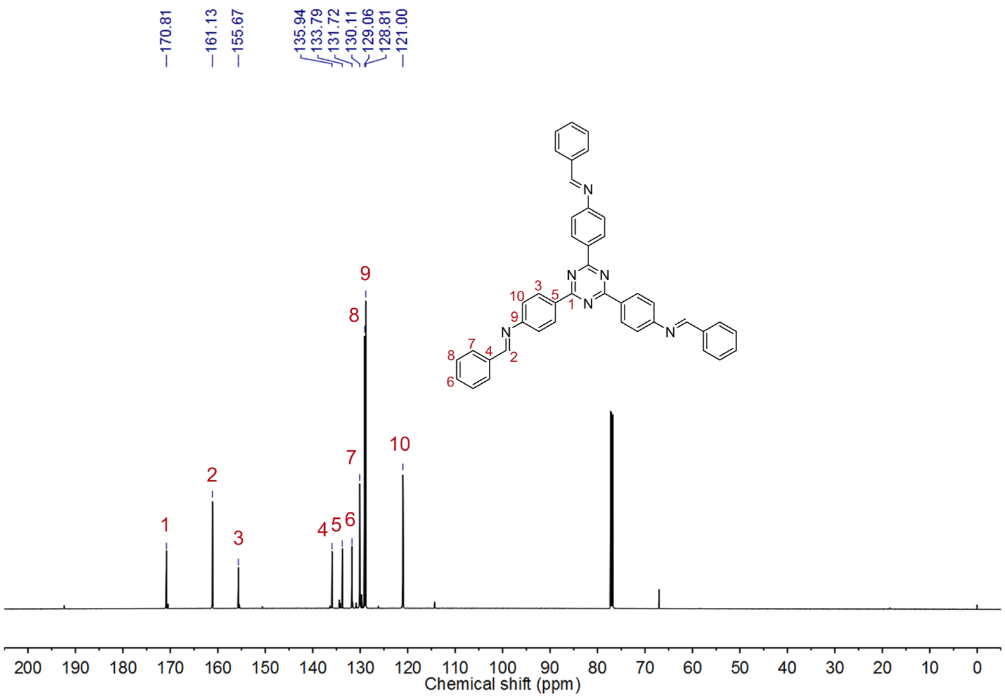

MC-TFPA 100 MHz, DMSO-d_6

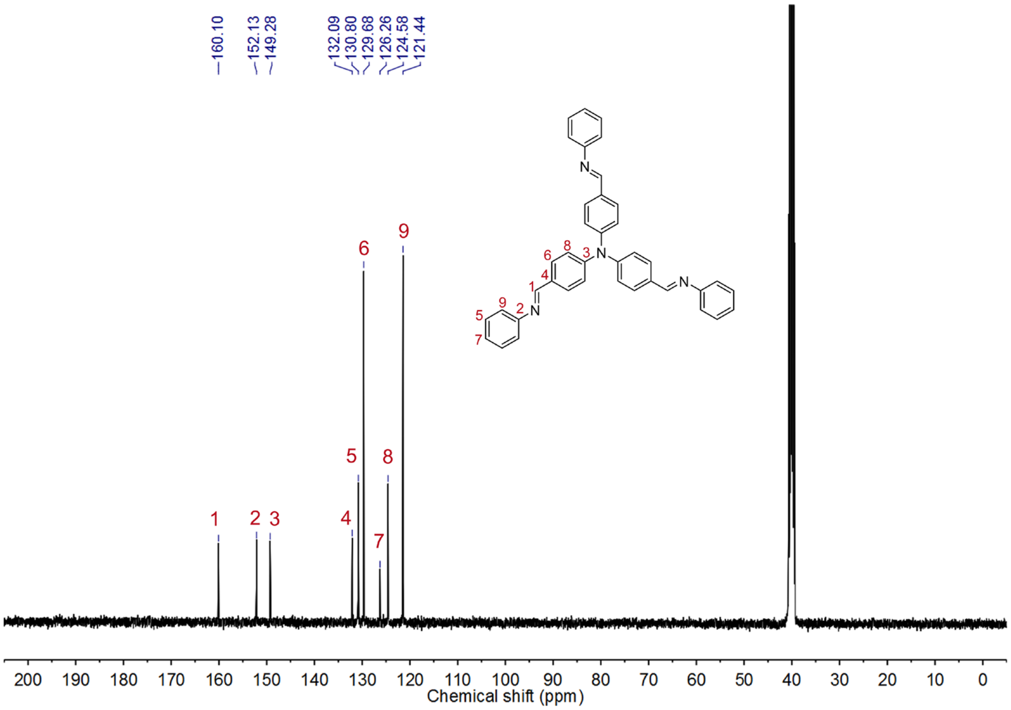

MC-TAPB 100 MHz, DMSO-d_6

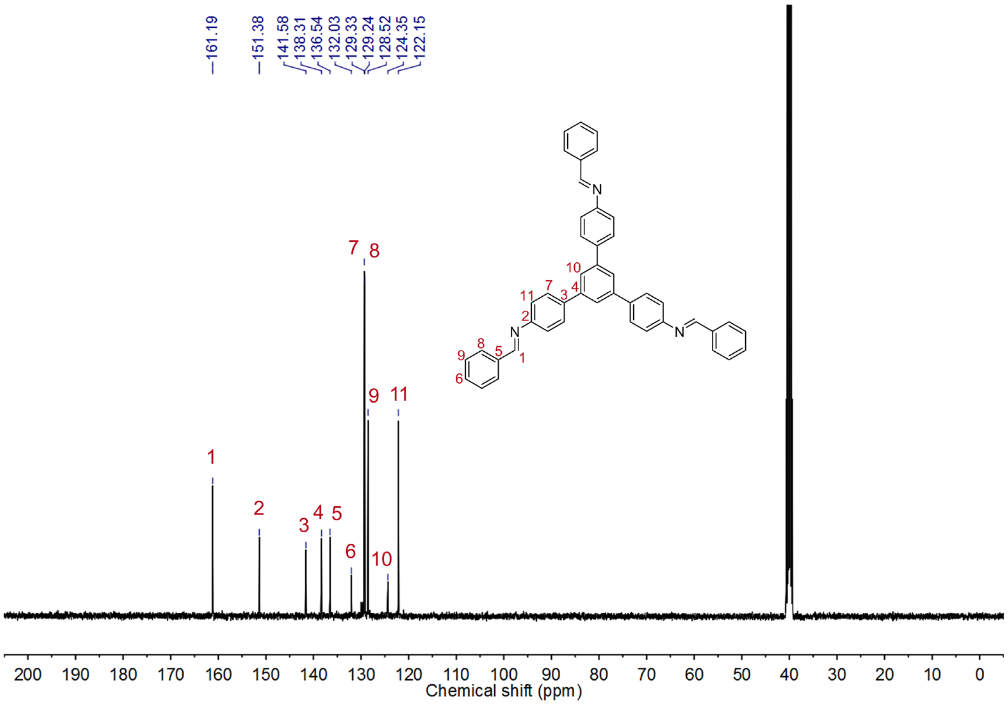

MC-TAPA 100 MHz, DMSO-d_6

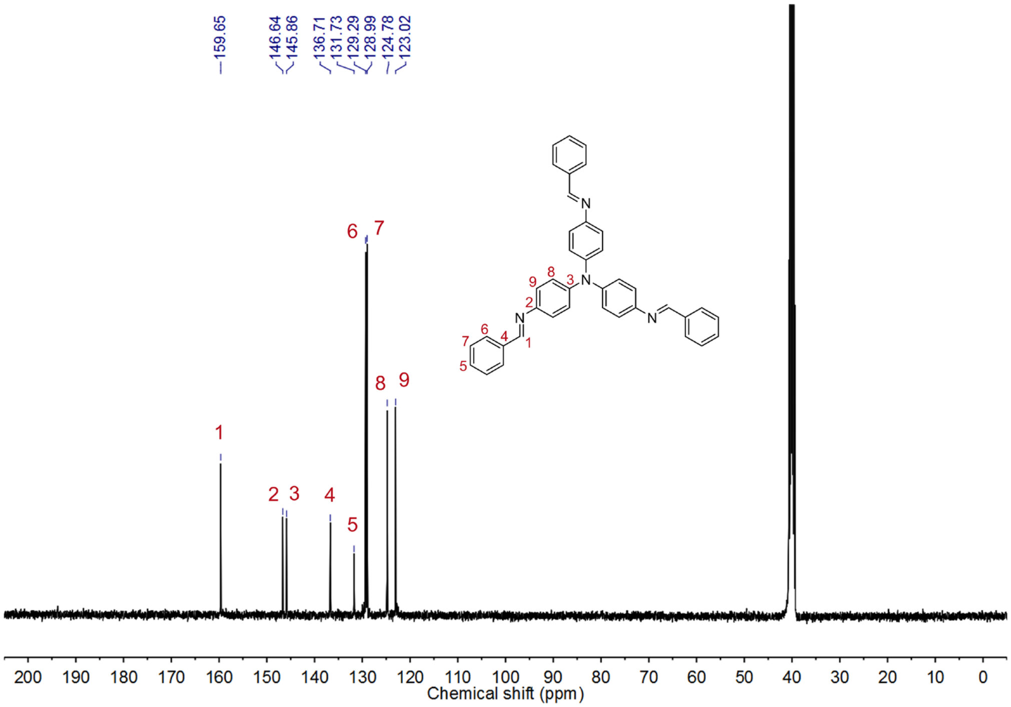

HRMS

MC-TAPT HRMS (ESI) calcd. for $C_{42}H_{31}N_6^+$ $[M+H]^+$ 619.2605, found 619.2616.

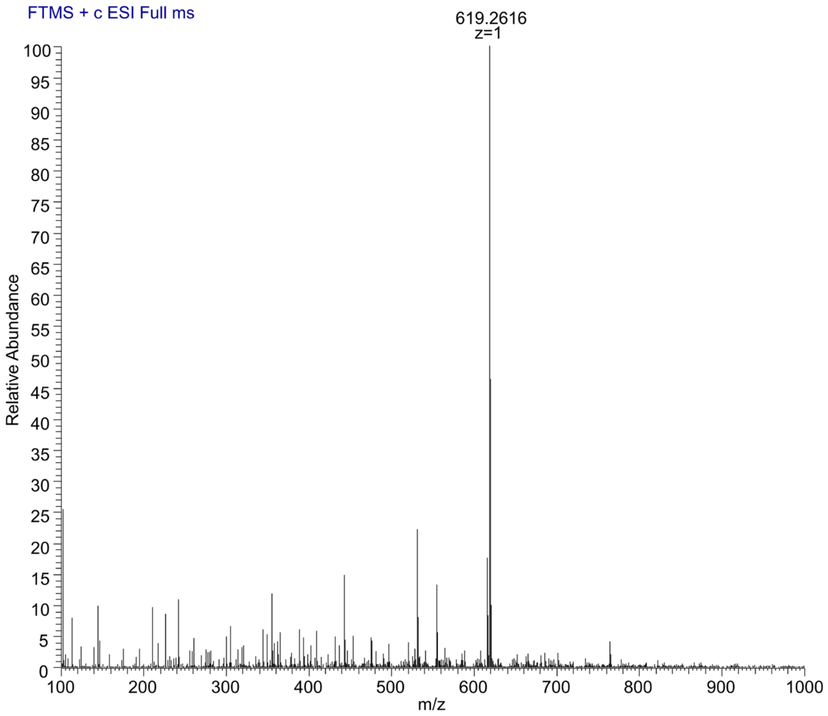

MC-TFPA HRMS (ESI) calcd. for $C_{39}H_{31}N_4^+$ [M+H]$^+$ 555.2543, found 555.2545.

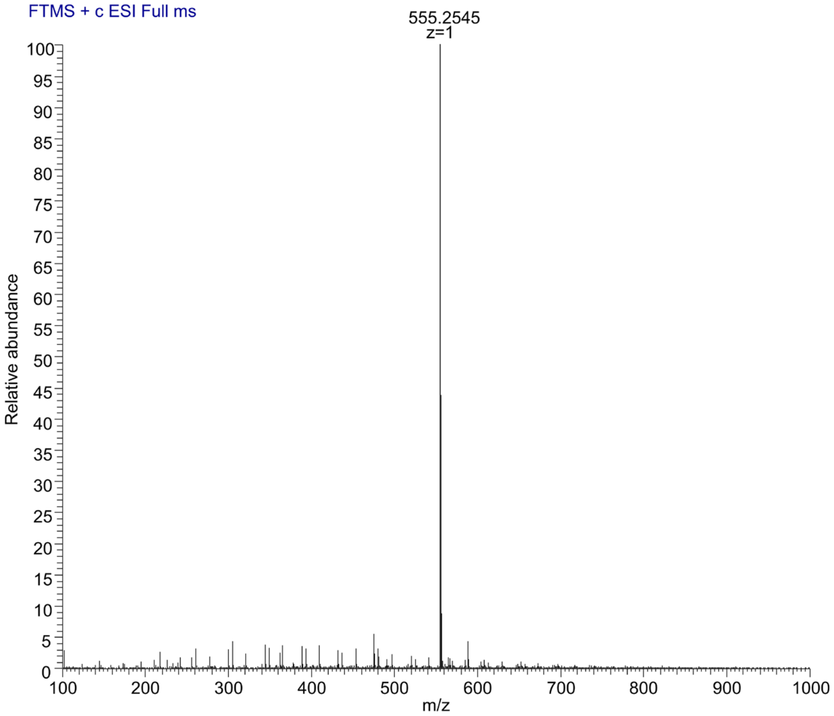

MC-TAPB HRMS (ESI) calcd. for $C_{45}H_{34}N_3^+$ [M+H]$^+$ 616.2747, found 616.2764.

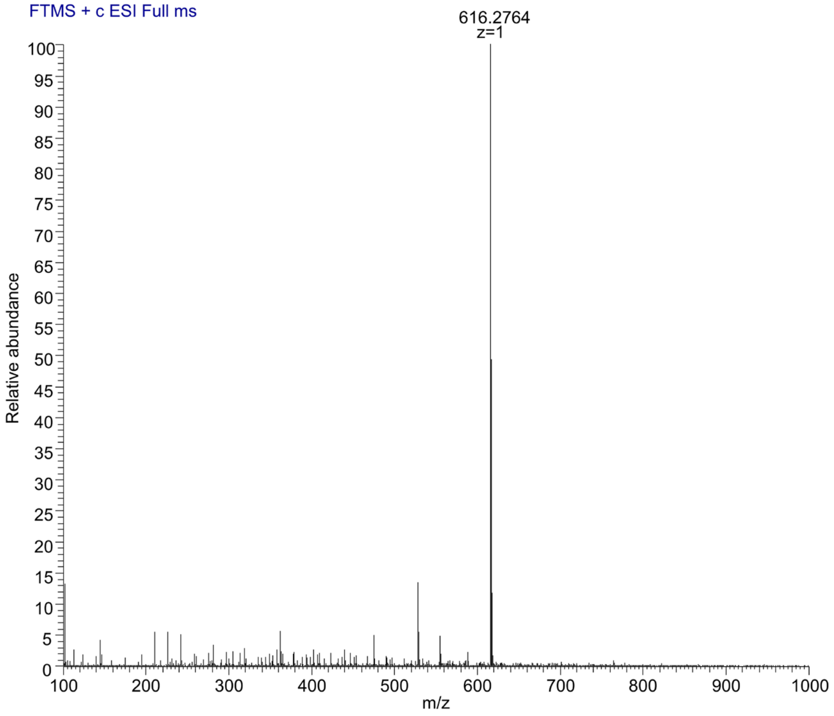

MC-TAPA HRMS (ESI) calcd. for $C_{39}H_{31}N_4^+$ [M+H]$^+$ 555.2543, found 555.2571.

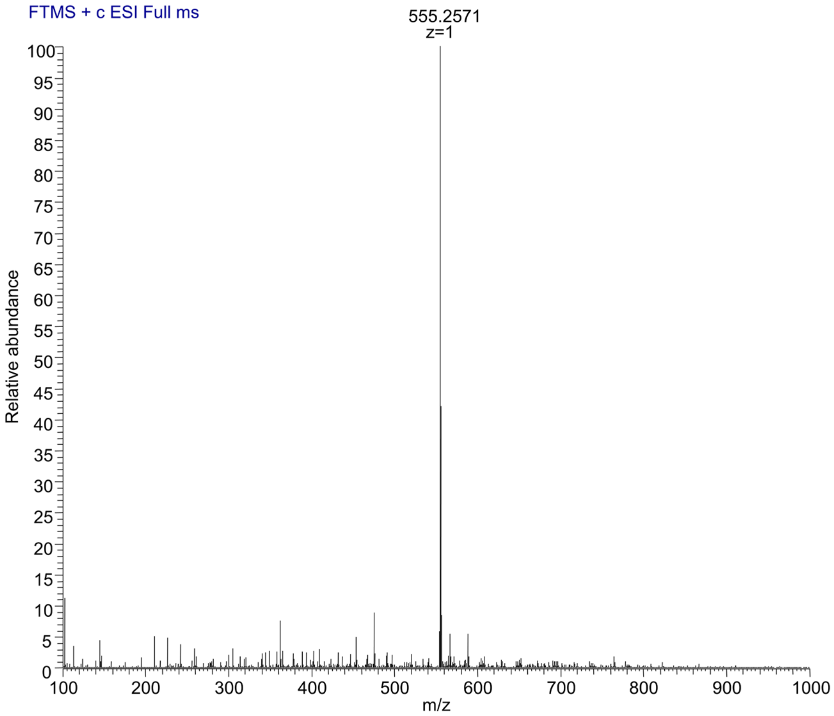

Supplementary References

1 Wang, N. et al. Dual intramolecular electron transfer for in situ coreactant-embedded electrochemiluminescence microimaging of membrane protein. *Angew. Chem. Int. Ed.* **60**, 197–201 (2021).

2 Wang, N. et al. Dual resonance energy transfer in triple-component polymer dots to enhance electrochemiluminescence for highly sensitive bioanalysis. *Chem. Sci.* **10**, 6815–6820 (2019).

3 Grigoras, M. & Stafie, L. Synthesis and characterization of linear, branched and hyperbranched triphenylamine-based polyazomethines. *Des. Monomers Polym.* **12**, 177–196 (2009).

4 Peng, Y. et al. Ultrathin two-dimensional covalent organic framework nanosheets: preparation and application in highly sensitive and selective DNA detection. *J. Am. Chem. Soc.* **139**, 8698–8704 (2017).

5 Bardeen, J. & Shockley, W. Deformation potentials and mobilities in non-polar crystals. *Phys. Rev.* **80**, 72–80 (1950).
